# Seamless lateral graphene p–n junctions formed by selective in situ doping for high-performance photodetectors

**DOI:** 10.1038/s41467-018-07555-6

**Published:** 2018-12-05

**Authors:** Gang Wang, Miao Zhang, Da Chen, Qinglei Guo, Xuefei Feng, Tianchao Niu, Xiaosong Liu, Ang Li, Jiawei Lai, Dong Sun, Zhimin Liao, Yongqiang Wang, Paul K. Chu, Guqiao Ding, Xiaoming Xie, Zengfeng Di, Xi Wang

**Affiliations:** 1 State Key Laboratory of Functional Materials for Informatics, Shanghai Institute of Microsystem and Information Technology, Chinese Academy of Sciences, 865 Changning Road, Shanghai, 200050 P.R. China; 2 Department of Microelectronic Science and Engineering, Faculty of Science, Ningbo University, Ningbo, 315211 P.R. China; 3 International Center for Quantum Materials, School of Physics, Peking University, Beijing, 100871 P.R. China; 4 State Key Laboratory for Mesoscopic Physics, School of Physics, Peking University, Beijing, 100871 P.R. China; 5 Materials Science and Technology Division, Los Alamos National Laboratory, Los Alamos, New Mexico 87545 USA; 6 Department of Physics and Department of Materials Science and Engineering, City University of Hong Kong, Tat Chee Avenue, Kowloon, Hong Kong P.R. China

## Abstract

Lateral graphene p–n junctions are important since they constitute the core components in a variety of electronic/photonic systems. However, formation of lateral graphene p–n junctions with a controllable doping levels is still a great challenge due to the monolayer feature of graphene. Herein, by performing selective ion implantation and in situ growth by dynamic chemical vapor deposition, direct formation of seamless lateral graphene p–n junctions with spatial control and tunable doping is demonstrated. Uniform lattice substitution with heteroatoms is achieved in both the boron-doped and nitrogen-doped regions and photoelectrical assessment reveals that the seamless lateral p–n junctions exhibit a distinct photocurrent response under ambient conditions. As ion implantation is a standard technique in microelectronics, our study suggests a simple and effective strategy for mass production of graphene p–n junctions with batch capability and spatial controllability, which can be readily integrated into the production of graphene-based electronics and photonics.

## Abstract (web summary)

Fabricating lateral graphene p–n junctions with controlled doping levels is instrumental to realize ultrafast and efficient optoelectronic devices. Here, the authors report a seamless graphene based photodetector doped by selective ion implantation and in-situ chemical vapour deposition.

## Introduction

Graphene with unique properties has large potential in atomic-level electronic/photonic systems^1–3^. To realize the full potential of graphene, fabrication of graphene p–n junctions is vital as they are the basic building blocks of modern semiconductor devices such as diodes, bipolar transistors, photodetectors, light-emitting diodes, and photovoltaic cells^4–6^. However, current techniques to produce graphene p–n junctions mainly based on multiple electrostatic gates^4,7–11^, charge transfer from adsorbed chemical dopants^12–15^, and electrostatic substrate engineering^16^, all are difficult to implement in practical applications. In contrast, substitutional doping by introducing dopants into the graphene lattice during chemical vapor deposition (CVD) is an effective means to mass produce doped graphene with the desirable carrier types (i.e., p-type or n-type)^17–20^ and tunable Fermi energy levels (i.e., dopant concentration)^21–23^. Using CVD doping, vertical graphene p–n junctions can be created by transfer and stacking of graphene with the desired doping type. However, the fabrication process has severe limitations including contamination and stacking orientation control which must be addressed^24,25^. Alternatively, a modulation-doped CVD strategy has been proposed to create lateral graphene heterostructures including i–n junctions^26^ and p–n junctions^6^. As the lateral graphene p–n junctions are formed by grafted growth of N-doped graphene from randomly nucleated B-doped graphene, it is challenging to obtain p–n junctions with good spatial control. In this respect, ion implantation which is routinely used in silicon processing to precisely dope bulk semiconductors offers excellent spatial and dose control. Recently, ion implantation has been extended to two-dimensional (2-D) materials including graphene for chemical doping and incorporation of N and B has been demonstrated^27–29^. However, the ion energy (e.g., <100 eV) much lower than the standard condition for IC industry is required to suppress the formation of implantation-induced defects. Owing to the monolayer or bilayer nature of graphene, the carbon atoms displaced during ion implantation with moderate energy cannot remain as interstitials within graphene basal plane and its immediate vicinities to participate in subsequent damage recovery through recombination of Frenkel pairs^30^. Therefore, the damage-curing annealing process routinely used in microelectronics processing cannot be applied directly to two-dimensional graphene.

Herein, we describe a well-controlled approach to fabricate seamless lateral graphene p–n junctions by selected-area pre-implantation of heteroatoms into the catalytic substrate followed by in situ growth of doped graphene by dynamic CVD. The doping type and dopant concentration are strictly determined by the pre-implanted heteroatom species and pre-designed fluence, respectively. Since the doping process is analogous to in situ doping of epitaxial semiconductor layers in the integrated circuits (IC) industry, both the p-type and n-type regions in the graphene p–n junction are expected to possess an excellent crystalline quality comparable to that of pristine graphene grown under identical conditions. Our strategy combines the substitutional doping advantage rendered by CVD and the spatial and dose control provided by ion implantation. The obtained lateral graphene p–n junctions exhibit a typical rectification behavior together with a superior photo-response rate. The photodetector fabricated on the lateral graphene p–n junction shows excellent performance in the range of 532–1550 nm with high detectivity (~10^12^ cmHz^1/2^ W^−1^) and responsivity (1.4~4.7 AW^−1^). As the ion implantation technology has been widely adopted in the current IC manufacturing, the fabrication of seamless lateral graphene p–n junctions with good spatial controllability, using the combination of ion implantation and in situ CVD, may pave the way for the industrial application of graphene in electronic and photonic devices.

## Results

### Fabrication of doped graphene and seamless lateral graphene p–n junction

**Fig. 1:**
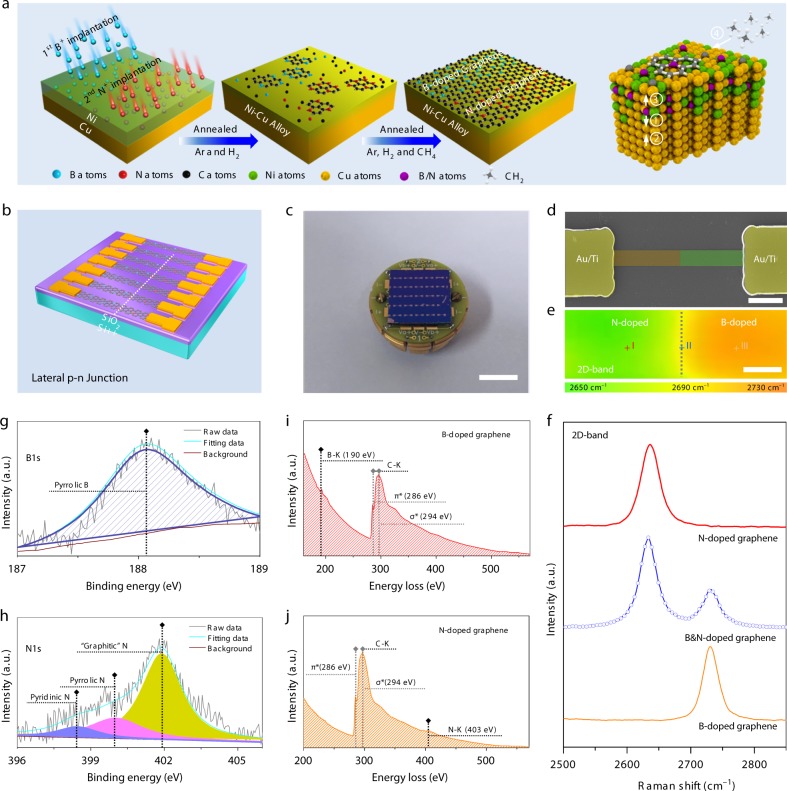
Characterization of the seamless lateral graphene p–n junction. **a** Schematic diagrams showing the synthesis process of the seamless lateral graphene p–n junctions. 1, 2, and 3 represent diffusion of Ni, Cu, and dopant (B or N) atoms, and 4 represents carbon atoms supplied by the decomposition of CH_4_. **b** Schematic diagram of the graphene photodetector array constructed on the seamless lateral graphene p–n junction. **c** A real image of the graphene photodetector array on the seamless lateral graphene p–n junction. The scale bar is 1 cm. **d** Pseudo-color SEM image of the seamless lateral graphene p–n junction device. The scale bar is 4 μm. **e** 2D peak map of the junction area of the lateral graphene p–n junction showing the B-doped graphene region (orange, B ion implantation using a fluence of 4 × 10^16^ atoms/cm^2^) and N-doped graphene region (green, N ion implantation with a fluence of 4 × 10^16^ atoms/cm^2^). The scale bar is 1 μm. **f** Raman spectra acquired from three different regions indicated in (**e**): (I) N-doped graphene portion (orange), (II) Junction location (blue), and (III) B-doped graphene region (red). **g** High-resolution XPS B-1*s* spectrum of the B-doped graphene film. **h** High-resolution XPS N-1*s* spectrum of the N-doped graphene film. EELS images of **i**, B-doped graphene and **j**, N-doped graphene

Figure 1a illustrates the synthesis process for the seamless lateral graphene p–n junctions. The Ni/Cu bilayered substrate is formed by deposition of a 300 nm thick Ni layer on the Cu foil, then B and N ions are successively implanted into the Ni/Cu bilayered substrate defined by photolithography to serve as dopant sources in the subsequent in situ CVD of graphene. The implantation energy is 60 keV to ensure that the dopants are distributed inside the top Ni layer and the ion implantation fluence is determined by the pre-designed doping level in graphene. Upon annealing in a mixture of H_2_ and Ar, inter-diffusion commences near the interface of Ni/Cu forming the Cu-like alloy^31^. Due to the limited solubility of B or N in the Cu-like alloy, the implanted B or N atoms gradually diffuse towards the top surface and participate into the growth of doped graphene after CH_4_ precursor is introduced into the annealing furnace. As the p-type and n-type graphene regions are defined by the prior photolithography, seamless lateral graphene p–n junction array is thus formed as expected. Distinct from the vertical p–n junction, no physical separation or boundary exists in our seamless lateral graphene p–n junction. The synthesis details are described in Supplementary Figures 1–2 and Supplementary Note I−1. The growth of doped graphene on the Cu-like alloy is self-limited to one atomic layer (Supplementary Figures 3–4 and Supplementary Note II). The graphene p–n junction array is transferred onto a 300 nm thick SiO_2_/Si substrate to fabricate the photodetector array (Fig. 1b). The corresponding optical image is depicted in Fig. 1c and the pseudo-color SEM image of one such seamless lateral graphene p–n junction device is displayed in Fig. 1d. Figure 1e shows the integrated position of the 2D band derived from Raman maps obtained from the seamless lateral graphene p–n junction revealing that the 2D band position changes from 2650 to 2730 cm^−1^ as the laser spot is moved from the N-doped graphene (green) to the B-doped graphene (orange). The micro-Raman spectra acquired along the p–n junction confirm that N shifts the 2D band to a smaller wavenumber, whereas B shifts it to a larger wavenumber relative to the pristine undoped graphene. At the junction between the N-doped and B-doped graphene, two characteristic bands corresponding to N doping and B doping emerge simultaneously, as shown in Fig. 1f. More details about the Raman scattering analysis of the N-doped and B-doped graphene are provided in Supplementary Figures 5–6 and Supplementary Note II–III. Figure 1g shows the deconvoluted high-resolution XPS spectrum of B-1*s* of the B-doped graphene and the slightly asymmetric B-1*s* peak suggests that the predominant bonding for B is B-C^32,33^. The deconvoluted high-resolution N-1*s* spectrum (Fig. 1h) shows three peaks at 398.2 eV, 399.9 eV, and 401.9 eV corresponding to pyrrolic N, pyridinic N, and graphitic N, respectively^34^. The prominent N-1*s* peak at 401.9 eV indicates that the majority of N atoms are substitutional. The chemical composition and bonding state in the N-doped and B-doped graphene are further analyzed by XPS survey and high-resolution XPS spectra of C-1*s* as shown in Supplementary Figures 7–8 and Supplementary Note IV. The presence of B or N in the graphene lattice is corroborated by electron energy-loss spectroscopy (EELS). In addition to the C-*K* edge^35^ (excitation of 1s carbon electrons to the π^*^ states ~286 eV and 1s to the σ^*^ states ~294 eV), the B-*K* edge at 190 eV^36^ and N-*K* edge at 403 eV^37^ are observed from the B-doped and N-doped graphene, respectively. The EELS elemental maps show that either B or N is uniformly distributed throughout graphene as described in Supplementary Figure 9 (Supplementary Note V) and extensive selected area electron diffraction (SAED) patterns (Supplementary Figures 10–11 and Supplementary Note VI) suggest that the doped graphene possess a considerable domain size^38^.

### Controllable doping in the synthesis of B-doped and N-doped graphene

**Fig. 2:**
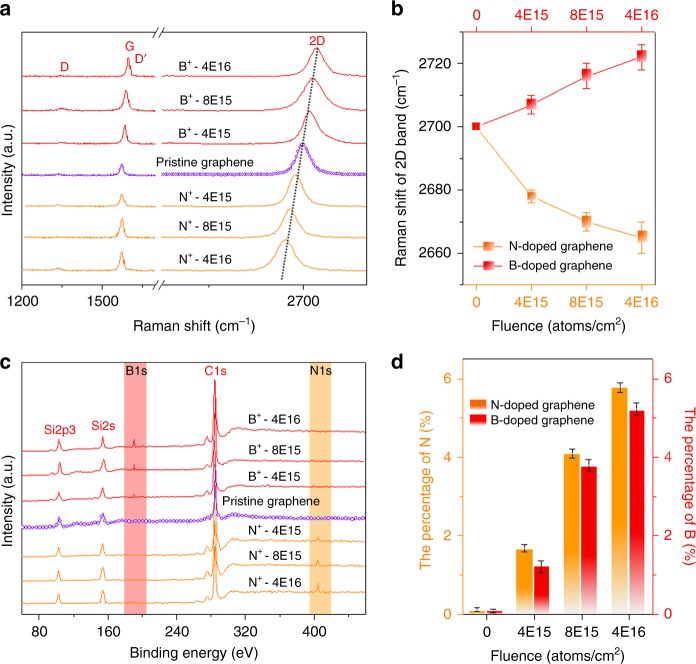
Control of doping in the synthesis of B-doped and N-doped graphene. **a** Raman spectra of the B-doped and N-doped graphene fabricated using different ion implantation fluences of B and N. **b** Shift in the 2D bands of B-doped and N-doped graphene as a function of ion implantation fluence. **c** XPS spectra of B-doped and N-doped graphene fabricated using different ion implantation fluences of B and N. **d** Histogram showing the atomic percentages of B and N in B-doped and N-doped graphene as a function of ion implantation fluence

In ion implantation, the dopant concentration can be controlled accurately. Figure 2a displays the representative Raman spectra of the B-doped graphene (red lines) and the N-doped graphene (orange lines) for different ion implantation fluences. Compared to the pristine graphene, B upshifts the 2D band and N downshifts it. As the implantation fluence is increased from 4 × 10^15^ to 4 × 10^16^ atoms/cm^2^, the 2D band of the B-doped graphene shifts gradually from ~2708 to ~2720 cm^−1^, whereas a monotonic decrease from ~2680 to ~2666 cm^−1^ is observed from the N-doped graphene (Fig. 2b). Electrochemical charging has been shown to be an efficient doping technique for graphene, although it may be difficult in practical device applications^39^. Hole doping with a positive potential upshifts the 2D band but electron doping downshifts the 2D band, which is in agreement with our in situ B and N doping described here. Since the G band always stiffens for doping levels below 0.6 eV^40^, the position of the G band always shifts to larger wavenumbers independent of the doping type, which is similar to doping graphene by electrochemical charging^39^. Figure 2c exhibits XPS spectra of B-doped and N-doped graphene synthesized with various implantation fluences of B and N, respectively. For the lightly doped graphene (fluence of 4 × 10^15^ atoms/cm^2^), both N-1*s* and B-1*s* signals begin to rise at ~ 400 eV and ~ 190 eV, respectively. As the ion implantation fluence is increased from 8 × 10^15^ to 4 × 10^16^ atoms/cm^2^, N-1*s*, and B−1*s* peaks become resolved and their intensity increase accordingly. Based on the XPS intensity, Fig. 2d displays the histogram of the doping level in the B and N-doped graphene as a function of ion implantation fluences. The B content in B-doped graphene can be tuned by the B fluences from 1.3 to 5.2% (estimated by XPS) and that of N-doped graphene from 1.8 to 5.6%. It should be noted the doping content can by tuned by the implantation fluence, but a saturation of the doping content does exist, as depicted in Supplementary Figure 12 and Supplementary Note VII.

### Structural, bandgap, and stability characterization of B-doped and N-doped graphene

**Fig. 3:**
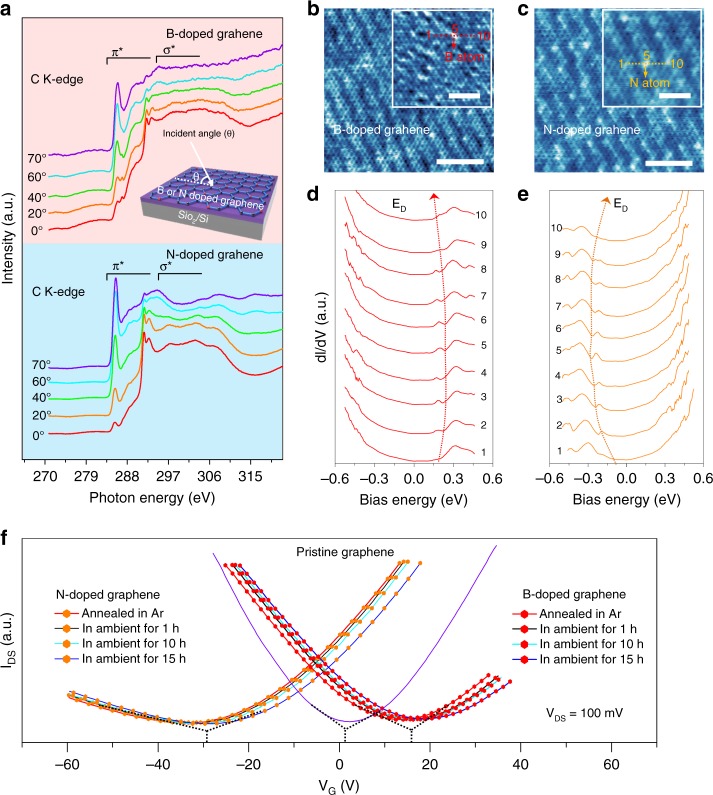
Structural, bandgap, and stability studies of doped graphene. **a** TEY mode C K-edge NEXAFS spectra of B-doped and N-doped graphene transferred onto the SiO_2_/Si substrate acquired at different incident angles (θ). The inset shows the schematic of the test setup in the angle-dependent NEXAFS experiments. STM topographical images of **b**, B-doped on a Cu-Ni alloy substrate (*V*_bias_ = −300 mV, *I*_set_ = 200 pA) [scale bar = 5 nm] and **c**, N-doped graphene on a Cu-Ni alloy substrate (*V*_bias_ = 300 mV, *I*_set_ = 200 pA) [scale bar = 5 nm]. The inset in each figure displays the selected region at a higher magnification [scale bar = 1 nm]. **d**, **e** Representative STS spectra along the dashed arrows in the inset (**b**, **c**), showing the characteristic density of states in B-doped (*V*_bias_ = −300 mV, *I*_set_ = 300 pA) and N-doped graphene (*V*_bias_ = 300 mV, *I*_set_ = 300 pA). The shift in the Dirac point is tracked by the red (orange) dotted line to show charge doping on the B (N) atoms. **f** Doping stability assessment of the B-doped and N-doped GFETs under ambient conditions for various time durations. N-doped and B-doped graphene synthesized by B and N ion implantation with the fluence of 4 × 10^16^ atoms/cm^2^

Strong evidence that the B and N atoms are incorporated in the planar graphene lattice is obtained by the near-edge X-ray absorption fine structure (NEXAFS)^41^ as shown in the inset in Fig. 3a. Figure 3a shows angle-dependent NEXAFS measured in total electron yield (TEY) mode at the C K-edges for B-doped (doping level of 5.2%) and N-doped (doping level of 5.6%) graphene, respectively. For both samples, the C K-edge NEXAFS spectra show strong peaks at ∼285 eV and ∼292 eV corresponding to the 1s to π^*^ and 1s to σ^*^ transitions, respectively. The sharp excitonic features at 291.5 eV indicate the long-range order in the electronic structure in the B-doped and N-doped graphene. The angular dependence in the C K-edge NEXAFS spectra in Fig. 3a is similar to that of the pristine graphene (shown in Supplementary Figure 13a and Supplementary Note VIII), indicating that the crystalline quality is preserved after doping with dilute heteroatoms^42,43^. Moreover, the B and N K-edge NEXAFS spectra are similar to the C K-edge NEXAFS spectra showing the 1s → π^*^ and 1s → σ^*^ transitions (Supplementary Figures 13b–e and Supplementary Note VIII). However, on account of the small doping concentration, the angular dependence of either the B or N K-edge NEXAFS is barely detected. Therefore, other methods are needed to determine whether the dopants are localized within or oblique to the graphene basal plane as presented below.

To gain insights into the incorporation of dopants as well as the local electronic structure of the doped graphene, scanning tunneling microscopy (STM) and scanning tunneling spectroscopy (STS) are performed at 77 K on the doped graphene grown on Cu-like alloy^44^. The two representative STM topographic images obtained from B-doped and N-doped graphene show that the clear honeycomb lattice is interspersed with bright protrusions, which correspond to the substitutional dopants or the intrinsic defects are present as shown in Fig. 3b and Fig. 3c, respectively. Moreover, the dopant concentrations for doped graphene can be also deduced from STM images, as shown in Supplementary Figure 14 and Supplementary Note VIII, which agrees well with the estimation by XPS. To further identify the origin of protrusions and characterize how the B-dopants and N-dopants affect the local change in electronic structure, spatially resolved STS spectra across the protrusions, are acquired as indicated by the dashed arrows in the corresponding STM images, as shown in Fig. 3d, e. For B-doped graphene, when the STM tip is far from the dopant, only one minimum corresponding to the Fermi level (0 eV), which is typical for graphene and attributed to inelastic excitation of a phonon^45^, appears. As the STM tip moves towards the B dopant, the second minimum, i.e., a depression, emerges at the positive sample bias, which is ascribed to the Dirac point (*E*_D_ = *e*V_D_) of graphene. The Dirac point is located at the positive sample bias, which is above the Fermi level, suggesting that the graphene film is doped with holes^46^. In addition, a noticeable shift in the Dirac point towards a more positive sample bias with enhanced depletion is observed as the STM tip gets closer to the B dopant due to electronic coupling between the B dopant and its neighboring C atom and the maximum shift is observed at exactly the center of the B atom. The recurrence of the Dirac point shift as the STM tip moves away from the B dopant verifies that the Dirac point is associated with B and the shift in the Dirac point is induced by hole charge doping due to B. As for the N-doped graphene, besides the Fermi level (0 eV), the depression associated with the Dirac point is also observed but at a negative sample bias, suggesting that the graphene film is electron doped. Furthermore, due to the distinct STS spectra obtained from B-doped and N-doped graphene, it is concluded that the observed protrusions originate from the dopants, not the suspected defects. Because of the similar electronic coupling effect, the circuitous shift of the Dirac point location is also observed when the STM tip moves across the N-doped region^44,47^. The same dependence has been previously reported for graphene supported by individual water clusters on Au (111), where nanoscale water clusters introduce the strong doping of electrons in graphene and the doping level is controlled by the cluster size^48^.

To study the doping effect and stability, both the B-doped and N-doped graphene are fabricated into back-gated graphene field effect transistors (GFETs) and compared with pristine graphene as shown in Fig. 3f. For the pristine graphene, the *I*_DS_−*V*_G_ curve reveals a typical symmetry in the electron and hole conductance with the charge neutrality at nearly zero volt (Supplementary Figure 15 and Supplementary Note IX). With regard to the B-doped graphene, the charge neutrality point of the GFETs is at a positive gate voltage, indicating that it is light p-type. In addition, transport in the B-doped graphene exhibits an obvious asymmetry in electron and hole conduction, where the conductance of hole carriers is retained, while the electron conductance decreases as consistent with the p-type doping effect^33^. For the N-doped graphene, the charge neutrality point of the GFETs shifts to a negative gate voltage consistent with the n-type doping effect^49^. Furthermore, the asymmetry in electron and hole conduction becomes more significant resulting in strong suppression of hole conduction together with unaltered electron conduction. Highly reproducible transport characteristics with neglected shift of the neutrality point are obtained from GFETs built on the B-doped and N-doped graphene at room temperature under ambient conditions, even exposed to the ambient condition for 15 h (Supplementary Figures 16–17 and Supplementary Note IX). The excellent doping stability suggests that the heteroatoms are incorporated in the basal plane of honeycomb lattice and remain inert to the external environment. Meanwhile, the doped graphene possesses good carrier mobilities (Supplementary Figure 18 and Supplementary Note IX), which are comparable to that reported by others^34,50^.

### Performance of the photodetector constructed on the seamless lateral graphene p–n junction

**Fig. 4:**
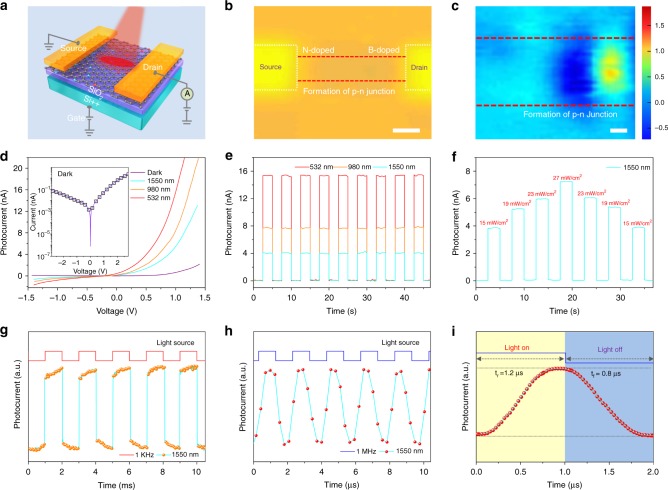
Photoelectric properties of the seamless lateral graphene p–n junction. **a** Schematic illustration of the photodetector. **b** Optical micrograph of the scanned area for the photocurrent mapping measurement with the dotted lines showing the contour of the photodetector. The scale bar is 2 μm. **c** Photocurrent mapping across the entire B-doped/N-doped seamless connected area (the fluences of 4 × 10^16^ atoms/cm^2^ are used for both N-doped and B-doped portions) at the biasing condition of *V*_ds_ = *V*_g_ = 0 V. The photoexcitation power is 500 μW and the wavelength is 633 nm. The laser spot size is 1 μm with the spatial resolution of 0.5 μm. The scale bar is 500 nm. **d** *I*_ds_−*V*_ds_ characteristics of the photodetector measured in the dark and under light illumination with variable wavelengths of 532, 980, and 1550 nm, respectively. The intensity of the illuminated light is fixed at 15 mW/cm^2^. The inset displays the dark current in logarithm scale. **e** Photo-switching behaviors of the photodetector towards the pulsed light illumination with variable wavelengths (*V*_ds_ = 1 V, *V*_g_ = 0 V). The light intensity is fixed at 15 mW/cm^2^. **f** Photo-response of the photodetector towards 1550 nm light illumination with variable intensity. Photo-response of the photodetector under the pulsed 1550 nm light illumination with frequencies of **g**, 1 kHz, and **h**, 1 MHz. **i** Enlarged photo-response curve at 1 MHz showing the rise time (*t*_r_) and fall time (*t*_f_)

Our approach which combines the substitutional doping advantage rendered by CVD and the spatial and dose control provided by ion implantation enables the formation of controlled p–n junctions suitable for photodetector arrays as illustrated in Fig. 1b, c and Fig. 4a, b (The fabrication details are described in Supplementary Note X). The dashed lines in Fig. 4b indicate the contour of the lateral graphene p–n junction connected by two electrodes. The photocurrent mapping in Fig. 4c clearly shows that the photocurrent response is localized to the narrow region between the two electrodes. As the position of graphene p–n junction interface coincides with the location of photocurrent (Supplementary Figure 19 and Supplementary Note XI), it suggests that the generation of photocurrent is due to the formation of p–n junction across the entire B-doped/N-doped area. Moreover, the photocurrent is also generated at graphene-metal contacts, and it attributes to the effect of photovoltaic (PV), in which the photogenerated charge carriers are accelerated to the electrodes by a built-in electric field^51–53^, as shown in Figure 19 and Supplementary Note XI, since good ohmic contact is formed and no significant Schottky barrier exists^54,55^ (Supplementary Figure 20 and Supplementary Note XI). The formation of p–n junction is further confirmed by the transfer characteristic curve obtained from FET made of graphene p–n junction, shown in Supplementary Figure 21 and Supplementary Note XI. It is found that the whole curve is divided into three regions denoted as n–n +, p–n and p +–p, and two maxima corresponding to the charge neutrality points (Dirac points) coexist, which is the hallmark of a graphene p–n junction^56,57^. To evaluate the performance of the individual photodetector in broadband detection, both visible light (e.g., green light (532 nm)) and near-infrared light (e.g., 980 and 1550 nm) with a fixed power density of 15 mW/cm^2^ are chosen to excite the p–n junction region. Figure 4d presents the *I*_ds_−*V*_ds_ characteristics of the lateral graphene p–n junction in the dark and under light illumination with different wavelengths. Importantly, a clear current rectification behavior, which is a typical feature for semiconductor p–n junction, is observed for the dark current, as shown in the inset of Fig. 4d. When the junction area is illuminated, a pronounced photocurrent is observed at the forward bias. In addition, the generated photocurrent increases rapidly as the wavelength is decreased from 1550 to 532 nm indicating the capability of photoelectric conversion. The photo-response of graphene p–n junction is further evaluated using the temporal light sources as depicted in Fig. 4e. The graphene p–n junction exhibits a distinct photo-response towards the light illumination with the wavelength varying from 532 to 1550 nm at zero bias. In addition, the corresponding photo-response of graphene p–n junction display the excellent reproducibility and stability. For pulsed light, the step rising and falling edges of the photocurrent suggest that the photo-induced voltage or photocurrent may be due to PV effect other than the bolometric effect^58^. However, PV effect should not be the dominant effect, since it usually leads to photocurrents in the same direction. While, as observed in Fig. 4c, the photocurrent switches sign as the raster-scanning of light moves from the B-doped portion to the N-doped portion of graphene p–n junction. It should be noted, similar to that of the PV effect, the sign of photothermoelectric (PTE) current in graphene p–n junction is usually unidirectional. But, it can be reversed in the unipolar junction regimes such as pp^+^ or nn−, as described previously^4,19,59–62^. Although the doping seems homogeneous across large area of doped graphene, the non-uniform doping still exists locally, especially near the p–n junction interface (Supplementary Figure 19 and Supplementary Note XI). Therefore, besides the possible PV effect, we believe that the observed of photocurrent with reversed polarity across graphene p–n junction is due to the PTE effect considering the formation of unipolar junction regimes due to the local non-uniform doping. The photodetector fabricated on the lateral graphene p–n junctions delivers high performance of 532 to 1550 nm with high detectivity (~10^12^ cmHz^1/2^ W^−1^) and responsivity (1.4~4.7 AW^−1^). Recently, high detectivity (~10^12^ cmHz^1/2^ W^−1^) is also achieved in the broad spectral range (300–950 nm) from the graphene p–n vertical-type tunnelling diodes, but the responsivity (0.4~1.0 AW^−1^) is inferior to our lateral graphene p–n junction^63^. The performance of photodetector built on graphene p–n junction in further evaluated by the pulsed light illumination (1550 nm) with variable intensity, as summarized in Fig. 4f. The photocurrent increases gradually as the intensity of light illumination increases from 15 to 27 mW/cm^2^, and decreases subsequently as the intensity attenuates back to 15 mW/cm^2^. It should be noted that the photocurrent barely varies under the light illuminations at a fixed intensity, which suggests the photodetector built on graphene p–n junction exhibit good repeatability. In addition, the good reproducibility of the photodetector device is also confirmed by the measurements from multiple devices, as summarized in Supplementary Figure 22 and Supplementary Note XII. Figures 4g, h show the photo-response of the seamless lateral graphene p–n junction photodetector to a pulsed 1550 nm laser with an oscilloscope sweeping frequency of 1 kHz and 1 MHz, respectively. The photodetector operates very well even illuminated by high-frequency (MHz) optical signals. Moreover, as shown in the enlarged photo-response curve at 1 MHz in Fig. 4i, the rise time (*t*_r_) and fall time (*t*_f_) are evaluated to be 1.2 and 0.8 μs, respectively. The response speed is superior to that reported previously from graphene-based photodetectors^64,65^. We believe that such an ultrafast response should be attributed to the lateral structure of our graphene p–n junctions at which fast transport of photo-generated carriers is maintained along the lateral direction due to the high in-plane mobility, which agrees with TCAD simulation results (Supplementary Figure 23 and Supplementary Note XIII).

## Discussion

Seamless lateral graphene p–n junctions are constructed by selective-area ion implantation of boron or nitrogen into the Ni/Cu bilayered substrate and subsequently in situ growth of the doped graphene by CVD. The dopant concentration in graphene is solely determined by the ion fluence of implantation. Our results demonstrate that the technique is able to incorporate the dopants into the graphene lattice and achieve uniform lattice substitution in both the boron-doped and nitrogen-doped regions. This seamless lateral graphene p–n junction exhibits superior photodetector performance compared to conventional graphene photodetectors. As the essential building blocks in photonic and electronic devices, the graphene p–n junction produced by the selective in situ doping approach is expected to facilitate the design and development of all-graphene devices especially transparent and flexible electronics and photonics.

## Methods

### Doped graphene synthesis

The Ni/Cu bilayer substrate was formed by the deposition of a 300 nm thick Ni layer on a high-purity Cu foil (25 μm, Alfa Aesar, item No. 46365) by an electron beam evaporator. Then, 60 keV B and N ions with different fluences were implanted into the selected areas specified by photolithography. The Ni/Cu bilayer substrates were firstly cleaned by acetone, isopropyl alcohol, and deionized water several times and then annealed at 950 °C for 30 min in Ar, H_2_, and CH_4_ (200:10:0.5 sccm flow rate) mix atmosphere. Finally, CH_4_ was shutoff and the sample was cooled down to room temperature in a mixture of Ar and H_2_ (200:10 sccm flow rate). The optimized thermal procedures are summarized in Supplementary Figures 1–2 and Supplementary Note I-1.

### Doped graphene transfer

The doped-graphene films were transferred onto an oxidized silicon substrate or quartz slides for analysis and characterization by a PMMA assisted wet transfer method. Detailed experimental procedures are provided in and Supplementary Note I-2.

### Characterization

Raman scattering (HORIBA Jobin Yvon HR800) was performed to evaluate the thickness, quality, and uniformity of the doped graphene films. Crystallographic information and the number of doped-graphene layers were determined by transmission electron microscopy (TEM) (FET-Tecnai G2F20 S-7WIN). The STM measurements were performed in the constant current mode by applying a bias voltage to the sample. The polarization dependence of the π- and σ-resonances of the graphene- and graphite-functionalized systems were investigated by NEXAFS at beamline 20A1 of National Synchrotron Radiation Research Center at Taiwan. XPS (PHI 5802, Physical Electronics Inc, Eden Prairie, MN) and EELS (FEI Tecnai G2 F20) were employed to determine the chemical composition of the doped graphene. The photoelectric measurements were conducted under ambient conditions using an Agilent (B1500A) semiconductor parameter analyzer together with the Keithley 4200 semiconductor characterization system.

## Electronic supplementary material

Supplementary Information

## Data Availability

The data that support the findings of this study are available from the corresponding author on reasonable request.
