# Supplementary material for: Seamless lateral graphene p–n junctions formed by selective in situ doping for high-performance photodetectors
Source: Nat Commun. 2018 Dec 5;9:5168. doi: 10.1038/s41467-018-07555-6 (PMC6281711; doi:10.1038/s41467-018-07555-6)
Supplement: Supplementary file 1 — Supplementary Information [file 41467_2018_7555_MOESM1_ESM.pdf]

Supplementary Information

Seamless lateral graphene p-n junctions formed by selective in situ doping for high-performance photodetectors

Gang Wang[1,2], Miao Zhang[1], Da Chen[1,2], Qinglei Guo[1], Xuefei Feng[1], Tianchao Niu[1], Xiaosong Liu[1], Ang Li[1], Jiawei Lai[3], Dong Sun[3], Zhimin Liao[4], Yongqiang Wang[5], Paul K. Chu[6], Guqiao Ding[1], Xiaoming Xie[1], Zengfeng Di[1,*] and Xi Wang[1]

[1] *State Key Laboratory of Functional Materials for Informatics, Shanghai Institute of Microsystem and Information Technology, Chinese Academy of Sciences, 865 Changning Road, Shanghai 200050, China*

[2] *Department of Microelectronic Science and Engineering, Faculty of Science, Ningbo University, Ningbo 315211, China*

[3] *International Center for Quantum Materials, School of Physics, Peking University, Beijing 100871, China*

[4] *State Key Laboratory for Mesoscopic Physics, School of Physics, Peking University, Beijing 100871, China*

[5] *Materials Science and Technology Division, Los Alamos National Laboratory, Los Alamos, New Mexico, 87545, USA*

[6] *Department of Physics and Department of Materials Science and Engineering, City University of Hong Kong, Tat Chee Avenue, Kowloon, Hong Kong, China*

* Correspondence to: zfdi@mail.sim.ac.cn (Z.F. Di)

Supplementary Note I. Materials and Methods

Supplementary Note I-1. Fabrication of doped graphene and seamless lateral graphene p-n junction

The fabrication process of the seamless lateral graphene p-n junction is schematically illustrated in Supplementary Figure 1. The Ni/Cu bilayered substrate was prepared by depositing a 300 nm thick Ni layer on a high-purity Cu foil (25 μm) using an electron beam evaporator. For doped graphene, the Ni/Cu bilayered substrates were implanted with 60 keV B or 60 keV N ions. To produce the seamless lateral graphene p-n junction, the Ni/Cu bilayered substrates were implanted with 60 keV B and 60 keV N ions in adjacent regions specified by conventional photolithography and the lift-off process. The implanted Ni/Cu sample was cut into 1×1 cm^2 pieces and placed at the center of the horizontal quartz tube. The quartz tube was evacuated to approximately 10^{-5} mbar and then 200 sccm argon (Ar, 99.9999% purity) and 10 sccm hydrogen (H_2, 99.9999% purity) were introduced until reaching atmospheric pressure. The samples were then heated to 950 ^{o}C and 0.5 sccm methane (CH_4, 99.9999% purity) was introduced to start the fabrication of doped graphene and seamless lateral graphene p-n junction. After deposition, CH_4 gas was shut off and the furnace was cooled to room temperature under flowing Ar (200 scm) and H_2 (10 sccm). The red box in the Supplementary Figure 1 shows the fabrication sequence of the seamless lateral graphene p-n junction by chemical vapor deposition (CVD) and the corresponding thermal process is summarized in Supplementary Figure 2.

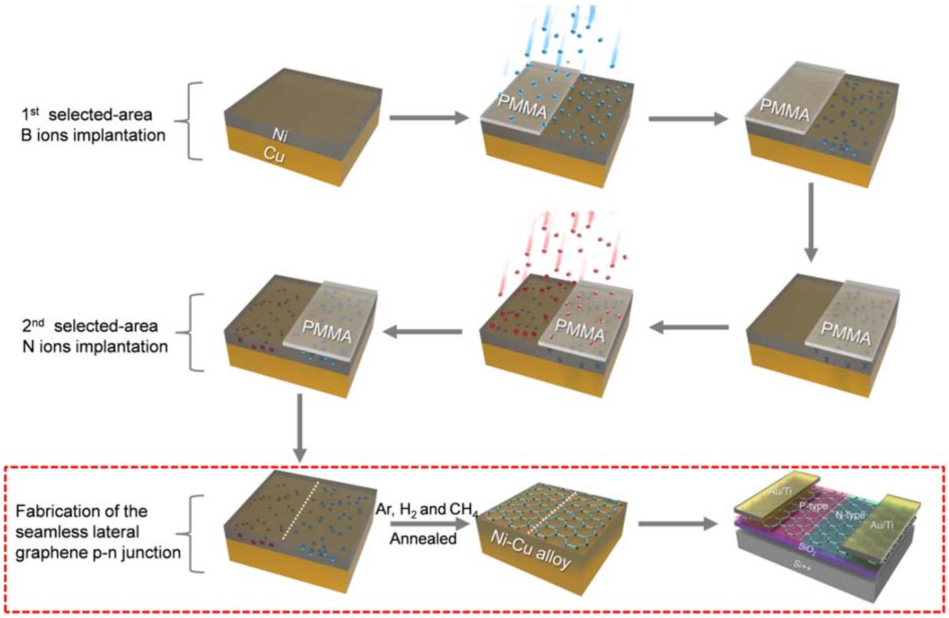

Supplementary Figure 1. Schematic diagrams showing the fabrication of the seamless lateral graphene p-n junctions. The red box shows the formation of the seamless lateral graphene p-n junctions by CVD.

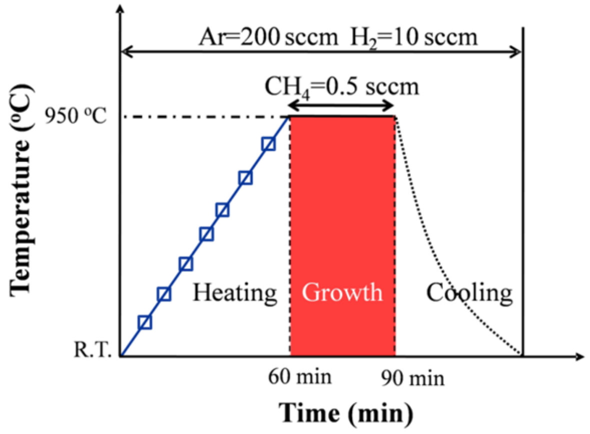

Supplementary Figure 2. Thermal process for the synthesis of the seamless lateral graphene p-n junction corresponding to the red box in Supplementary Figure 1.

Supplementary Note I-2. Transfer of doped graphene

For electrical characterization, the doped graphene films were transferred using the PMMA-assisted wet-transfer method. A thin layer of polymethyl methacrylate (MicroChem 950 PMMA C, 3% in chlorobenzene) was spin-coated onto the sample (2500 rpm for 30 s) to protect the doped graphene films and also provides the mechanical support. Afterwards, the Ni-Cu alloy was etched in an aqueous bath containing $FeCl_3$, H_2O, and concentrated HCl (3.5 g, 100 mL, and 10 mL, respectively) releasing the PMMA/graphene film which floated on top of the solution. After washing with deionized water, the PMMA/graphene films were transferred to the target substrate and annealed at 180 ^oC for 30 min to improve adhesion to the substrate. PMMA was dissolved gradually with acetone and deionized water and the graphene films were washed with isopropanol and dried with nitrogen (N_2) gas. Finally, the doped graphene films were annealed in Ar (200 sccm) and H_2 (50 sccm) for 8 h at 300 ^oC to remove trace PMMA residues.

Supplementary Note II. Crystalline quality and thickness uniformity of the B-doped and N-doped graphene

The doping effects in graphene were studied by Raman scattering spectroscopy. Supplementary Figure 3 depicts the Raman spectra of the B-doped graphene and N-doped graphene samples in comparison with pristine graphene. Three primary peaks, D band at 1350 cm^{-1}, G band at 1580 cm^{-1}, and 2D-band at 2700 cm^{-1} emerged. Compared with pristine graphene, the enhanced D-band and prominent D’-band appeared from both B-doped graphene and N-doped graphene due to defects caused

by in-plane doping of B and N atoms embedded in the graphene hexagonal lattice[1-2]. The Raman spectra of B-doped graphene and N-doped graphene show distinct band shifts. For B-doped graphene, the G-band blue-shifts by 14 cm^{-1} and the 2D-band blue-shifts by 8 cm^{-1}. For N-doped graphene, the G-band blue-shifts by ~15 cm^{-1} while the 2D-band red-shifts by 11 cm^{-1}. The direction of the 2D-band shift in N-doped graphene is opposite to that in B-doped graphene as consistent with the previous reports[3-4]. The number of graphene layers is determined by the intensity ratio of the 2D to G peak (I_{2D}/I_G). For the B-doped graphene and N-doped graphene, the I_{2D}/I_G ratios are larger than 1.5 indicating that only monolayered graphene films are formed.

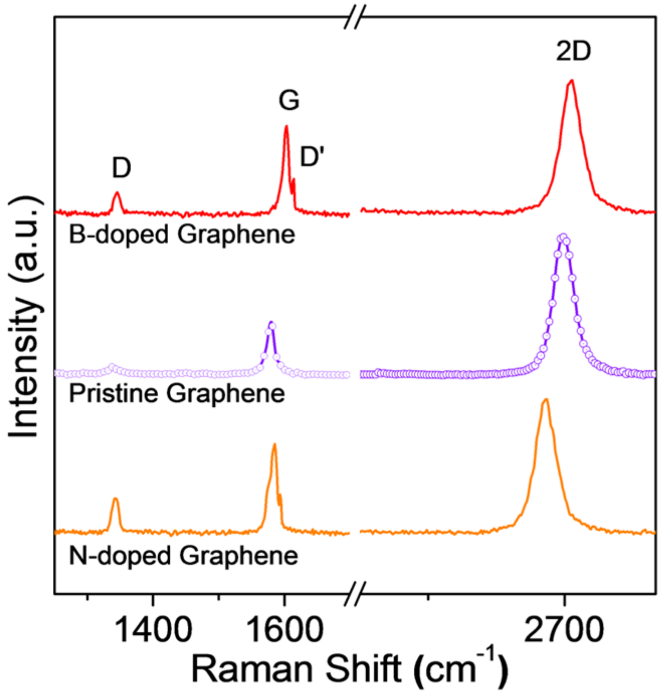

Supplementary Figure 3. Typical Raman spectra of the B-doped graphene (red line), pristine graphene (dashed purple line), and N-doped graphene (orange line) transferred to the SiO_2/Si substrate. The B/N doped graphene is synthesized by B/N

ion implantation with a fluence of 4×10^{16} atoms/cm^2.

To determine the thickness of the doped graphene, the transmittance at 550 nm was measured from the doped graphene films transferred to the quartz substrates as shown in Supplementary Figure 4. The optical transmittance decreases gradually as the ion implantation fluence of B or N is increased from 4×10^{15} to 4×10^{16} atoms/cm^2, but the value maintains no less than 96% at 550 nm. As the absorbance of monolayered graphene is 2.3%[5], it can be inferred that the doped graphene only has one layer regardless of the doping concentration[6]. The layer number in the doped graphene revealed by the transmittance measurement agrees with the intensity ratio of the 2D to G peak (I_{2D}/I_G) obtained by Raman scattering. The high optical transmittance also suggests that the doped graphene has immense potential in transparent electronics[7-8].

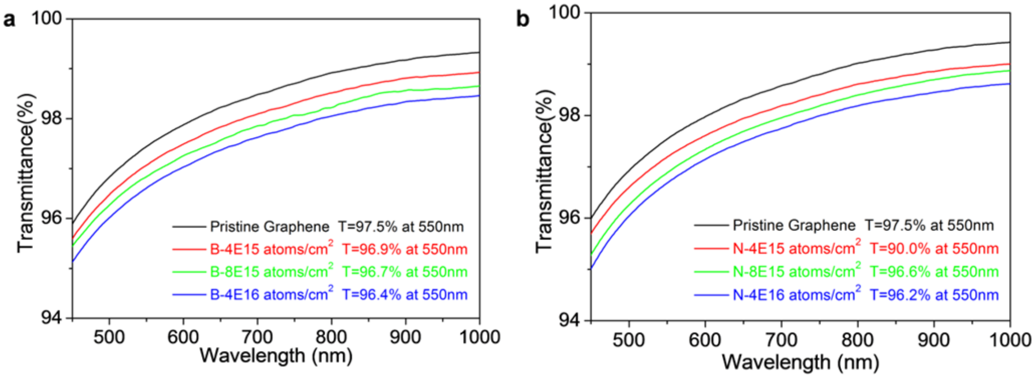

Supplementary Figure 4. Optical transmittance spectra of **a,** B-doped and **b,** N-doped graphene synthesized using different ion implantation fluences of B and N, respectively. The spectrum of the pristine graphene (black line) is also provided for comparison.

To determine the crystalline quality and thickness uniformity of the B-doped graphene and the N-doped graphene, the Raman maps of the band intensity ratios of I_D/I_G and I_{2D}/I_G over a 8 μm × 8 μm area with a step size of 0.2 μm are shown in Supplementary Figure 5. For the B-doped (Supplementary Figure 5a) and N-doped regions (Supplementary Figure 5b), the band intensity ratios of I_D/I_G are as low as ~0.1 across the region indicating that the two doped graphene films possess high crystallinity. The corresponding I_{2D}/I_G ratios are in the range of 1.5~2 suggesting formation of uniform monolayer graphene as shown in Supplementary Figure 5c and Supplementary Figure 5d. It should be noted that neither the crystalline quality nor the layer number of doped graphene films vary as the heteroatom changes.

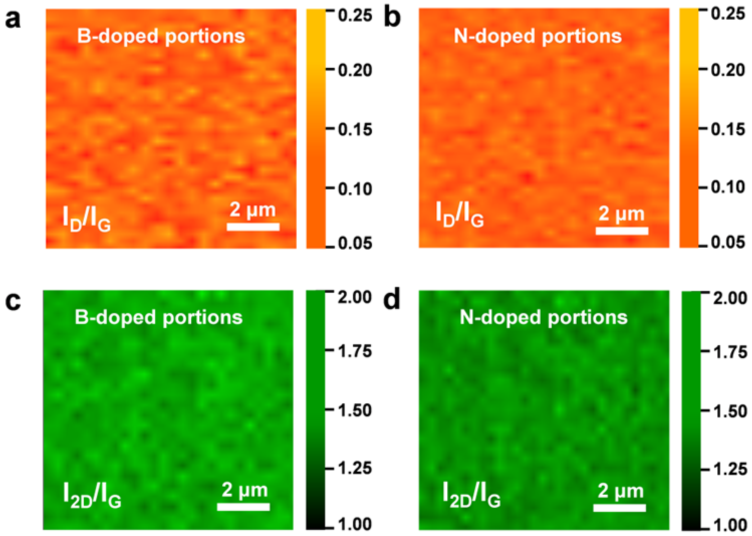

Supplementary Figure 5. Two-dimensional Raman maps of the I_D/I_G band intensity ratio for **a,** B-doped region and **b,** N-doped region of the p-n junction. Two-dimensional Raman maps of the I_{2D}/I_G band intensity ratio for **c,** B-doped region and **d,** N-doped region of the p-n junction. The B/N doped graphene is synthesized by B/N ion implantation with a fluence of 4×10^{16} atoms/cm^2.

Supplementary Note III. Doping uniformity of the synthesized B-doped and N-doped graphene

To determine the doping uniformity of graphene, Raman maps[9-10] were conducted on 8×8 μm^2 area with a step size of 0.2 μm as shown in Supplementary Figure 6. Compared to pristine graphene (Supplementary Figure 6a and Supplementary Figure 6g), for the B-doped graphene, the G-band blue-shifts to 1590 cm^{-1} (Supplementary Figure 6b) showing the presence of micrometer-sized patches with higher B concentration (green). And, the 2D-band blue-shifts to 2710 cm^{-1} (Supplementary Figure 6h) showing the presence of micrometer-sized patches with higher B concentration (pink) as well. The histograms of the G-band and 2D-band positions for the B-doped region are shown in Supplementary Figure 6e and Supplementary Figure 6k. More than 85% of the G-band and 2D-band blue-shift suggesting uniform B-doped graphene. With regard to N-doped graphene, the G-band blue-shifts to 1590 cm^{-1} (Supplementary Figure 6c), showing the presence of micrometer-sized patches with higher N concentration (green). The 2D-band red-shifts to 2690 cm^{-1} (Supplementary Figure 6i) indicating the presence of micrometer-sized patches with higher N concentration (gray). The direction of the 2D-band shift in the N-doped graphene is opposite to that of the B-doped region. The histograms of the G-band and 2D-band positions in the N-doped graphene show that more than 90% of the G-band blue-shift and 2D-band red-shift, indicating uniform N doping as shown in Supplementary Figure 6f and Supplementary Figure 6i.

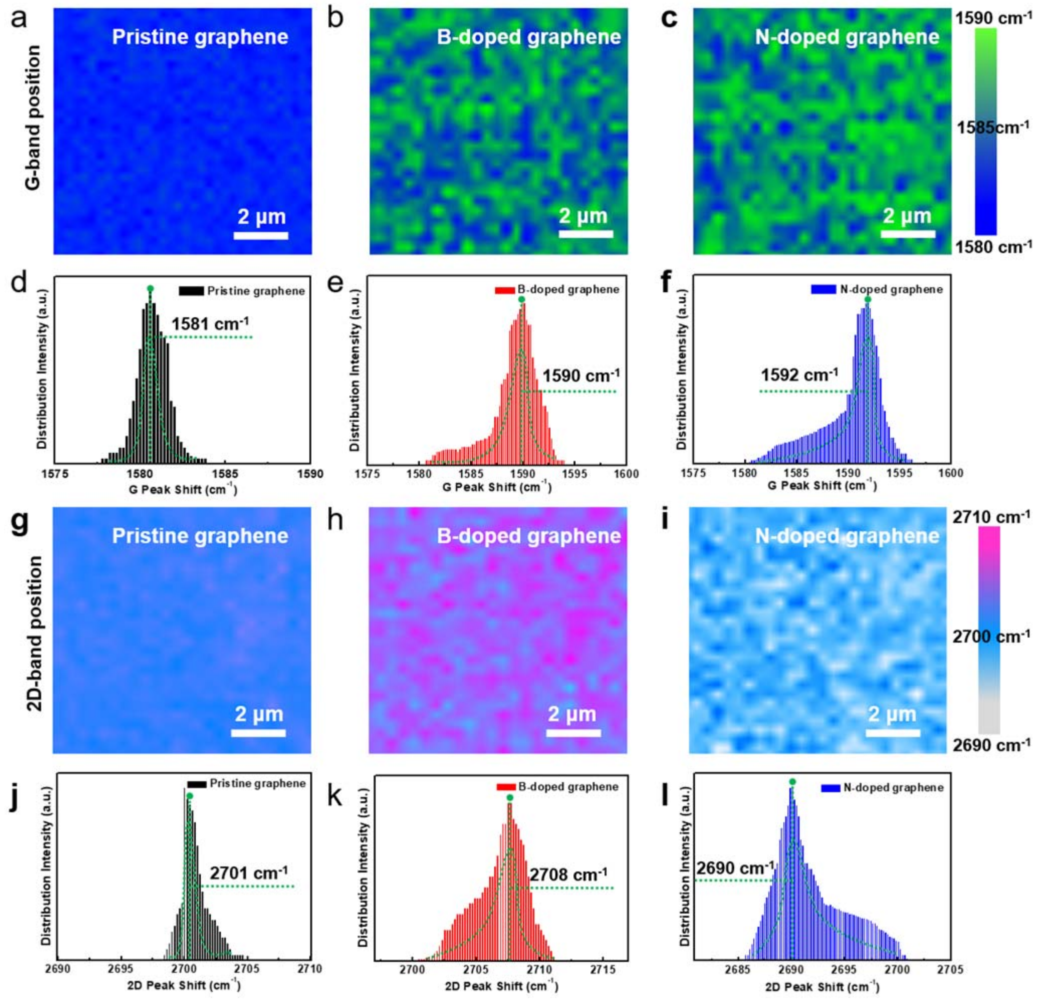

Supplementary Figure 6. Two-dimensional Raman maps of the G-band position for **a,** Pristine graphene, **b,** B-doped graphene, and **c,** N-doped graphene. G-band frequency histograms of **d,** Pristine graphene, **e,** B-doped graphene, and **f,** N-doped graphene from the maps in (a), (b) and (c). Two-dimensional Raman maps of the 2D-band position for **g,** Pristine graphene, **h,** B-doped graphene, and **i,** N-doped graphene. 2D-band frequency histograms of **j,** Pristine graphene, **k,** B-doped graphene, and **l,** N-doped graphene from the maps in (g), (h) and (i).

Supplementary Note IV. Chemical composition and chemical bonding states of

the B-doped and N-doped graphene

XPS was performed to determine the chemical composition and states. As shown in Supplementary Figure 7, for pristine graphene, no meaningful B and N signals are observed. For B-doped graphene, the B signal appears near 190.9 eV suggesting B atoms exist in graphene[11]. For N-doped graphene, the pronounced peak near at 400 eV is attributed to the N 1*s* state[12].

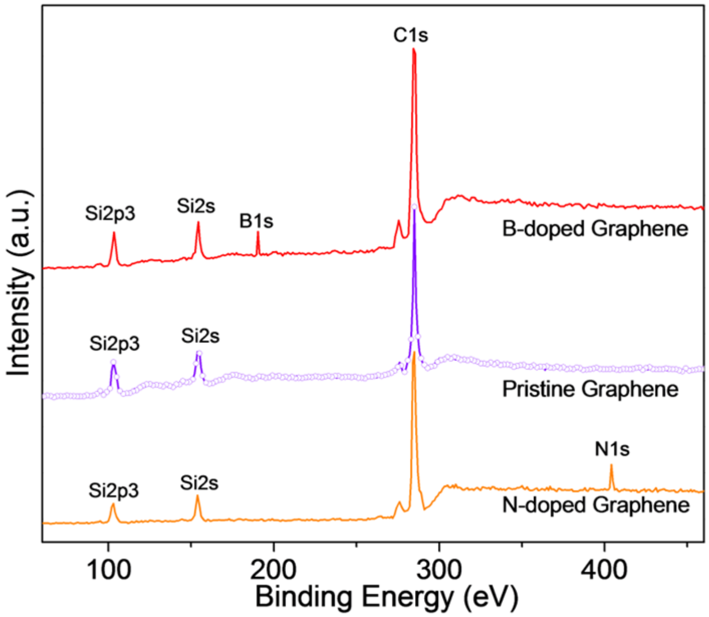

Supplementary Figure 7. Survey XPS spectra of the B-doped graphene (red), N-doped graphene (orange), and pristine graphene (dashed purple line) transferred onto the SiO_2/Si substrate. The B/N doped graphene is synthesized by B/N ion implantation with a fluence of 4×10^{16} atoms/cm^2.

Supplementary Figure 8 shows the deconvoluted high-resolution XPS spectra of C-1*s* for B-doped graphene, N-doped graphene, and pristine graphene. For doped graphene, the C 1*s* signal broadens and becomes more asymmetrical[12]. For pristine

graphene, the prominent C 1*s* peak at 284.3 eV corresponds to the graphite-like sp^2 hybridized state and the FWHM of the C 1*s* band is close to 0.56 eV corresponding to undoped and highly crystalline sp^2 carbon[13]. For B-doped graphene and N-doped graphene, the FWHM of the C 1*s* band increases to 0.98 eV and 0.77 eV, respectively. The increased asymmetry and broadening are attributed to localized lattice disorder arising from changes in the bonding configuration, especially sp^2 to an sp^3 after B and N doping[12]. The C 1*s* peak of B-doped graphene can be split into two, i.e., the C=C at ~283. 8 eV and the C=B at 283.1 eV. The C 1*s* peak of N-doped graphene can be fitted with three components, i.e., the C=C at 284.7 eV, the C=N at 285.5 eV, and the C-N at 286.6 eV, respectively.

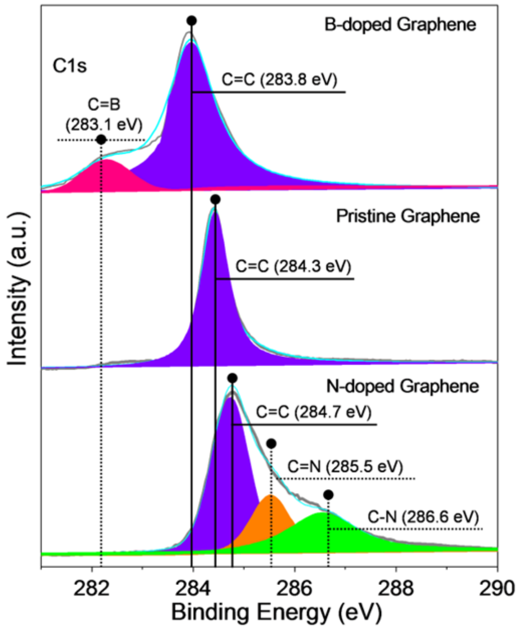

Supplementary Figure 8. Deconvoluted high-resolution XPS C-1*s* spectra of the B-doped graphene, N-doped graphene, and pristine graphene.

Supplementary Note V. Transmission electron microscopy (TEM), selected-area

electron diffraction (SAED) patterns, and electron energy-loss spectroscopy (EELS) characterization of the doped graphene.

The plan-view TEM images in Supplementary Figure 9a1 and Supplementary Figure 9a2 indicate that both B-doped graphene and N-doped graphene are continuous over a large area. The high-resolution TEM (HR-TEM) images acquired randomly from edges (Supplementary Figure 9b1 and Supplementary Figure 9b2) reveal that the doped graphene films are monolayered. As shown in the insets in Supplementary Figure 9a1 and Supplementary Figure 9a2, the SAED patterns exhibit only one set of hexagonal diffraction pattern which suggests that a single-crystal lattice exists in the field of view. For B-doped graphene, the uniform distributions of C and B across the graphene film are confirmed by the EELS elemental maps in Supplementary Figure 9c1 and Supplementary Figure 9d1, respectively. With regard to the N-doped graphene, the uniform distributions of C and N across the doped graphene film are also revealed in Supplementary Figure 9c2 and Supplementary Figure 9d2, respectively.

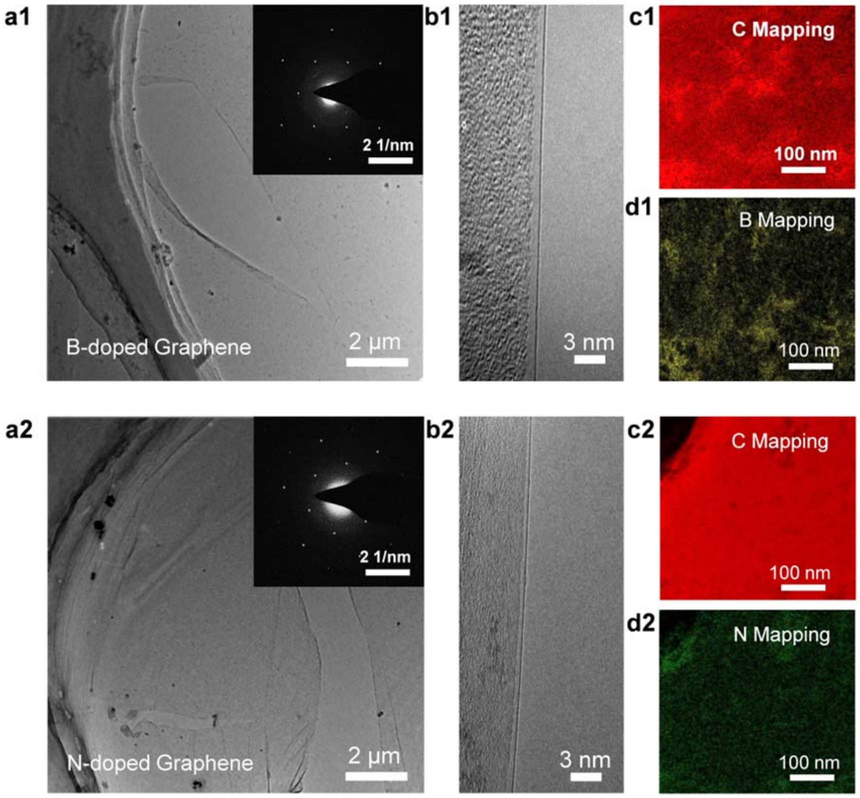

Supplementary Figure 9. TEM, HR-TEM, and SAED patterns and EELS maps of B-doped and N-doped graphene. **a1,** and **a2,** Plan view TEM images showing the continuity of the B-doped and N-doped graphene films, respectively. The inset in each figure shows the corresponding SAED pattern. **b1,** and **b2,** Cross sectional HR-TEM image showing that the B-doped and N-doped graphene are monolayered. **c1-d1,** EELS elemental maps of C and B in the B-doped graphene, respectively. **c2-d2,** EELS elemental maps of C and N in the N-doped graphene, respectively.

Supplementary Note VI. Crystallographic information of B-doped and N-doped graphene

The plan-view TEM images in Supplementary Figure 10a and Supplementary Figure 11a indicate that both the B-doped and N-doped graphene films are continuous

over a large area. For the B-doped (Supplementary Figure 10b) and N-doped (Supplementary Figure 11b) graphene films, the SAED patterns exhibit only one set of hexagonal diffraction pattern and the band intensity ratio (outermost to innermost) of ~1:2 (Supplementary Figure 10c and Supplementary Figure 11c) suggests the formation of single-layer doped graphene[14]. To investigate the spatial crystallographic orientation of the doped graphene, SAED patterns are collected at different locations and marked in Supplementary Figure 10a and Supplementary Figure 11a. For the B-doped graphene film, most of the SAED patterns (Supplementary Figures 10 1-7) show the same set of hexagonal diffraction spots without rotation, indicating the presence of a considerable size of the B-doped graphene domain. The rather small variation in relative rotation of $<\pm 3^{o}$ range observed in several SAED patterns (Supplementary Figures 10 2-5) near the edge of the measured area may be ascribed to local bending of the B-doped graphene film during the measurement. For the N-doped graphene films, most of the measured areas (Supplementary Figures 11 1-7) show the preferential crystal orientation within a $\pm 2^{o}$ range as well[15].

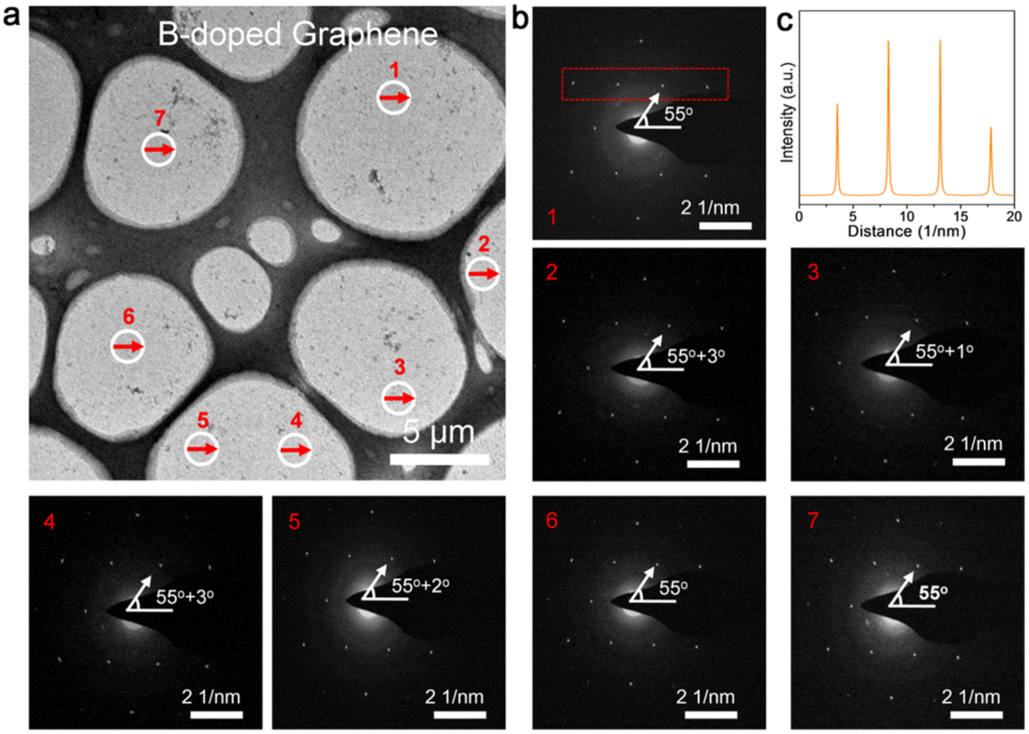

Supplementary Figure 10. TEM and SAED patterns of the B-doped graphene film. **a,** Plan-view TEM images showing the continuity of the B-doped graphene film over a large area. The numbered white circles indicate different locations where the SAED patterns are obtained. The colored arrows represent the orientation of the B-doped graphene grains in the selected locations normalized to that at location 1. **b**, SAED patterns of the B-doped graphene. (c) Profiles of the diffraction spot intensities along the box in (b). The number (1-7) in each SAED pattern indicates the acquired location in (a). The B doped graphene is synthesized by B ion implantation with a fluence of 4×10^{16} atoms/cm^2.

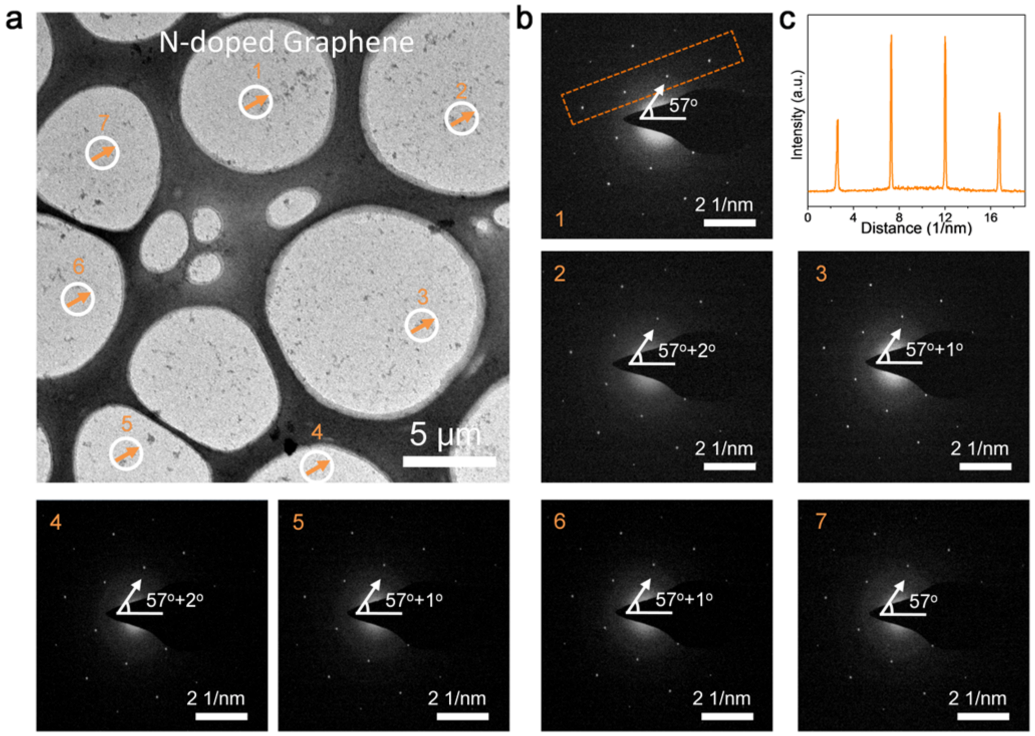

Supplementary Figure 11. TEM and SAED patterns of the N-doped graphene film. **a,** Plan-view TEM images showing the continuity of the N-doped graphene film across a large area. The numbered white circles indicate different locations where SAED patterns are obtained. The colored arrows represent the orientation of the N-doped graphene grains in the selected locations normalized to that at location 1. **b,** SAED patterns of the N-doped graphene. **c,** Profiles of the diffraction spot intensities along the box in (b). The number (1-7) in each SAED pattern indicates the acquired location in (a). The N doped graphene is synthesized by N ion implantation with a fluence of 4×10^{16} atoms/cm^2.

Supplementary Note VII. Saturation behavior of B-doped and N-doped graphene

As the implantation fluence is increased from 4×10^{15} to 8×10^{16} atoms/cm^2, the 2D band of the B-doped graphene shifts gradually from ~2708 to ~2720 cm^{-1},

whereas a monotonic decrease from ~2680 to ~2666 cm^{-1} is observed from the N-doped graphene (Supplementary Figure 12a-b). Note that the implantation fluence is increased from 4×10^{16} to 8×10^{16} atoms/cm^2, the 2D band of the B-doped and N-doped graphene are almost impossible to shifts. As the ion implantation fluence is increased from 4×10^{15} to 8×10^{16} atoms/cm^2, N-1*s* and B-1*s* peaks become resolved and their intensity increase accordingly (Supplementary Figure 12c). Based on the XPS intensity, Supplementary Figure 12d displays the histogram of the doping level in the B and N-doped graphene as a function of ion implantation fluences. The B content in B-doped graphene can be tuned by the B fluences from 1.3% to 5.2% (estimated by XPS) and that of N-doped graphene from 1.8% to 5.6%. However, due to saturation behavior, the implantation fluence is increased from 4×10^{16} to 8×10^{16} atoms/cm^2, the doping content of the B/N-doped graphene are almost unchanged.

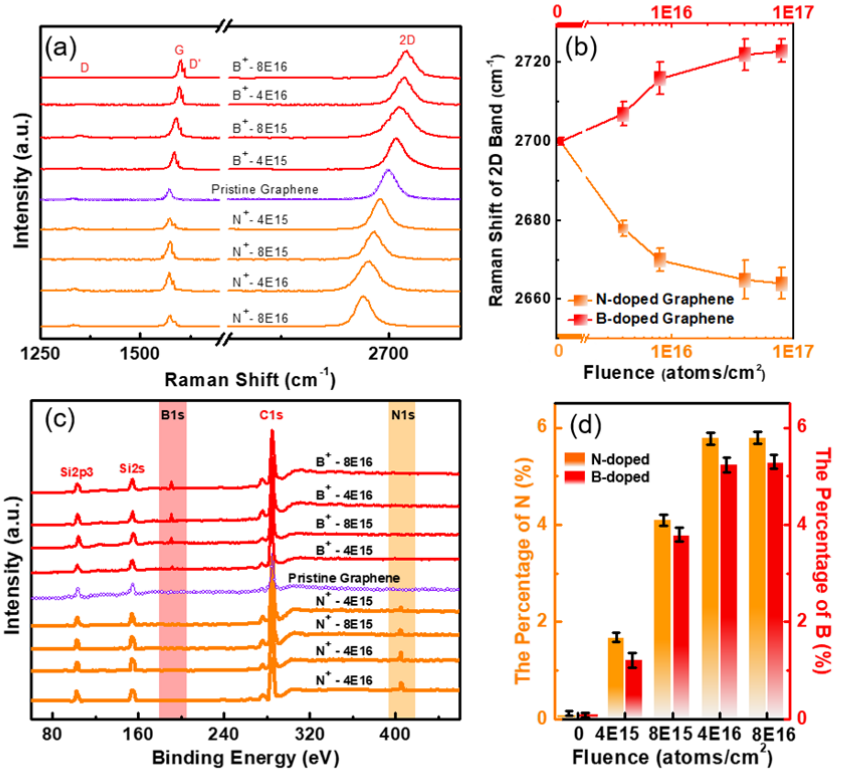

Supplementary Figure 12. a, Raman spectra of the B-doped and N-doped graphene fabricated using different ion implantation fluences of B and N (from 4×10^{15} to 8×10^{16} atoms/cm^2). **b,** Shift in the 2D bands of B-doped and N-doped graphene as a function of ion implantation fluence. **c**, XPS spectra of B-doped and N-doped graphene fabricated using different ion implantation fluences of B and N (from 4×10^{15} to 8×10^{16} atoms/cm^2). **d,** Histogram showing the atomic percentages of B and N in B-doped and N-doped graphene as a function of ion implantation fluence.

Supplementary Note VIII. NEXAFS and STM of B-doped graphene and N-doped graphene

Supplementary Figure 13 shows angle-dependent NEXAFS measured in the total electron yield (TEY) mode at the C, B and N K-edges. For pristine graphene, the features at 285.5, 291.8, and 292.7 eV can be attributed to the transitions from the C 1s core level to the π^*, excitonic and σ^* states of graphene[16], respectively, as shown in Supplementary Figure 13a. Supplementary Figure 13b and Supplementary Figure 13c show the TEY mode angle-dependent B and N K-edge NEXAFS spectra from the pristine graphene. None of distinct peaks appears indicating that no B or N atoms are distributed in the pristine graphene as expected. The B K-edge spectra of B-doped graphene and the N K-edge spectra of N-doped graphene are shown in Supplementary Figure 13d, S13e, showing the $1s\rightarrow\pi^*$ and $1s\rightarrow\sigma^*$ transitions[17-18]. The angular dependence of the B K-edges and N K-edges is quite similar to that of the C K-edges, indicating that the features are due to planar bonds in a 2D system.

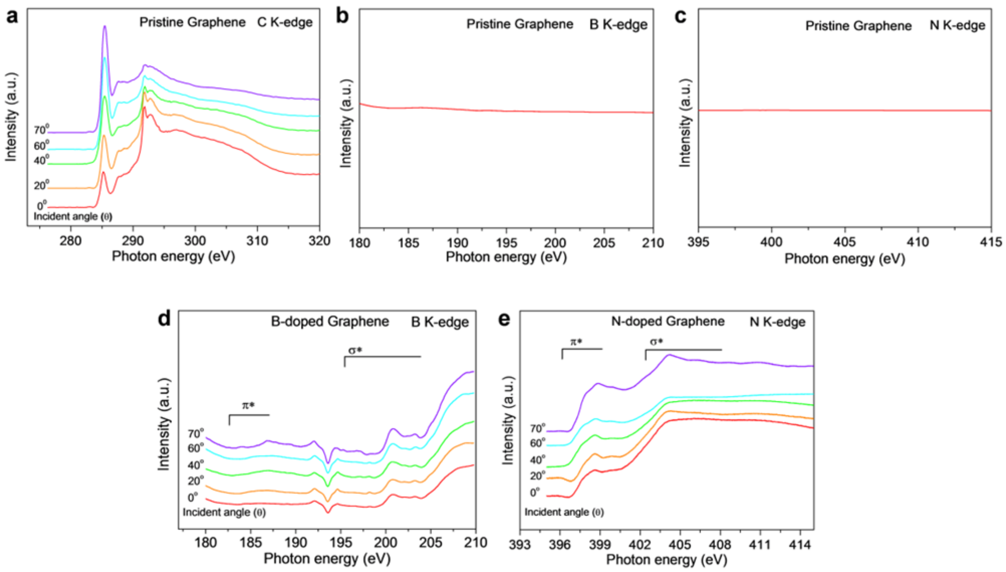

Supplementary Figure 13. a, TEY mode C K-edge NEXAFS spectra of pristine graphene at different incident angles. **b,** TEY mode B K-edge NEXAFS spectra of the pristine graphene. **c,** TEY mode N K-edge NEXAFS spectra of the pristine graphene. **d,** TEY mode B K-edge NEXAFS spectra of the B-doped graphene. **e,** TEY mode N K-edge NEXAFS spectra of the N-doped graphene.

We provided the detailed analysis of dopant concentration for doped graphene from STM images, as shown in Supplementary Figure 14a and Supplementary Figure 14b. To better observe the atomic structure of doped graphene, STM images with higher magnification are provided as the insets in Supplementary Figure 14a and Supplementary Figure 14b. Two insets have the similar size of 2×2 nm^2. Considering the areal density of carbon atoms in graphene is 3.82×10^{15} cm^{-2},[19] there are 153 carbon atoms distributed across the whole inserted image. By simply counting up the number of bright protrusions, there are about 8 N or B atoms distributed in the area of 2×2 nm^2. Therefore, the dopant concentrations for N-doped graphene and B-doped

graphene are about 8/153 (5.2%), which agrees well with the estimation by XPS.

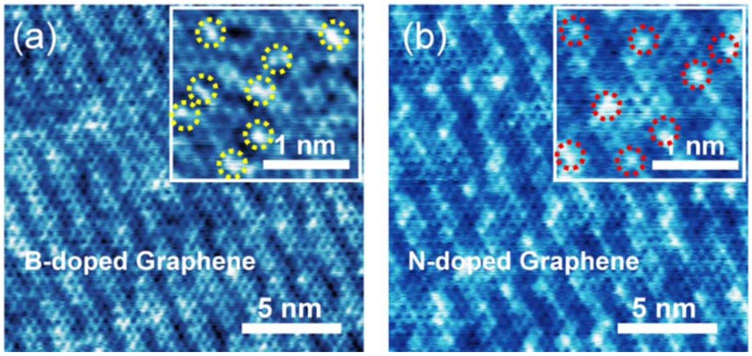

Supplementary Figure 14. STM topographical images of **a,** B-doped graphene on a Cu-Ni alloy substrate (V_{bias} = -300 mV, I_{set} = 200 pA) and **b,** N-doped graphene on a Cu-Ni alloy substrate (V_{bias} = 300 mV, I_{set} = 200 pA). The inset in each figure corresponds to the selected region with a higher magnification.

Supplementary Note IX. Electrical properties of B-doped and N-doped graphene

The electronic transport properties of the B-doped and N-doped graphene are evaluated using the back-gated graphene field effect transistors (GFETs). The doped graphene and pristine graphene were transferred to a highly doped p-type Si substrate coated with a 300 nm thick thermal oxide followed by deposition of the source and drain electrodes with Au/Ti (50/10 nm) by electron beam evaporation. Afterwards, another photolithographic step employing inductively coupled plasma (ICP) was used to pattern the doped graphene and pristine graphene to form a field-effect transistor with a channel length of 8 μm and width of 2 μm. To improve the contact on the back-gated GFETs device, annealing was performed in Ar (500 sccm) and H_2 (10 sccm) at 300 ^{o}C for 8 h. The back-gated GFETs were characterized under ambient conditions using an Agilent (B1500A) semiconductor parameter analyzer. Highly

reproducible transport characteristics (I_{DS}-V_G) were obtained from GFETs at room temperature. The typical I_{DS}-V_G curve acquired at a V_{DS} of 100 mV shows that both hole and electron conduction can be achieved by gating, as shown in Figure 3(f) in the main manuscript. As for the pristine graphene, the neutrality point of the GFETs is located at a gate voltage of V_G near at 0 V (Supplementary Figure 15a). The output characteristics of the pristine graphene is shown in Supplementary Figure 15b. The linear I_{DS}-V_{DS} behavior indicates good ohmic contact between the Ti/Au contact and pristine graphene channels[20].

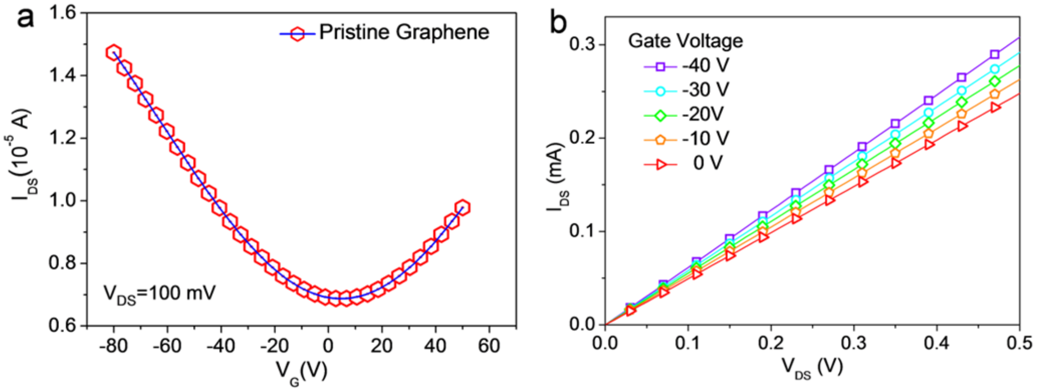

Supplementary Figure 15. a, Transfer (I_{DS}-V_G) curves of the pristine GFET at V_{DS} =100 mV. **b,** Output (I_{DS}-V_{DS}) curves of the pristine GFET at different V_G.

Supplementary Figure 16 displays the histogram of the neutrality point distribution of the GFETs fabricated on the B-doped graphene and N-doped graphene synthesized by B and N ion implantation with fluence of 4×10^{16} atoms/cm^2. For the B-doped graphene, the histogram for a total of 16 devices demonstrates that the neutrality points of the GFETs are at a positive gate voltage of V_G = ~15 V, indicating that graphene is slightly hole-doped. For the N-doped graphene, the histogram of

the neutrality point distribution for the 16 devices shows that the neutrality points of the GFETs shift towards a negative gate of V_G = ~-25 V, which is consistent with the electron doping effect.

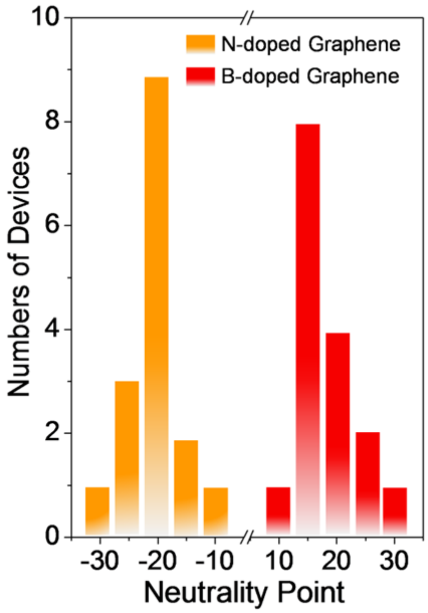

Supplementary Figure 16. Histogram of neutrality point distribution of the GFETs fabricated on the doped graphene.

The doping stability of the B-doped graphene and N-doped graphene devices was studied by measuring the neutrality points of the GFETs exposed to ambient air for different time durations, as shown in Supplementary Figure 17. A negligible shift is observed with increased exposure period to ambient air from both the B-doped and N-doped GFETs and the electronic transport properties of the GFETs are almost unchanged even after exposure to ambient air for 15 h, indicating that the dopants are incorporated stably in the honeycomb lattice plane of graphene.

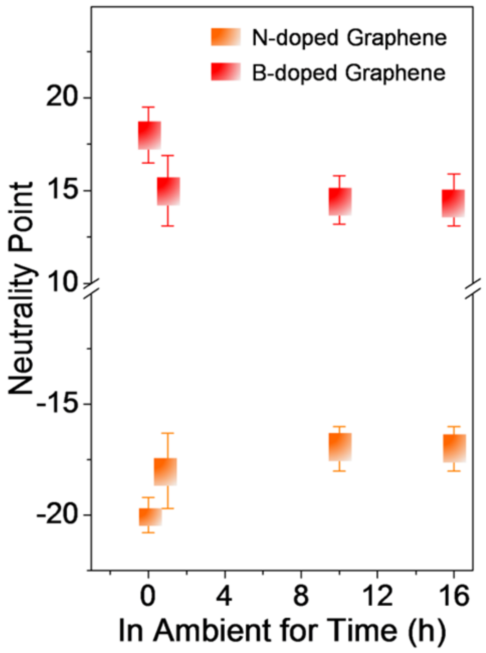

Supplementary Figure 17. Neutrality point of the GFETs fabricated on doped graphene as a function of exposure duration to ambient air.

The doped graphene films were transferred to highly doped p-Si substrate with a 300 nm thick thermal oxide, followed by deposition of source and drain electrodes with Au/Ti (50/10 nm) by electron beam evaporation. Afterwards, another photolithographic step employing inductively coupled plasma (ICP) was used to pattern the graphene to form a field-effect transistor with a channel length of 8 μm and width of 2 μm. To impove the contact of the back-gated GFETs device, annealing was performed in Ar (500 sccm) and H_2 (10 sccm) at 300 ^{o}C for 8 h in a tube furnace. The back-gated GFETs were characterized under ambient conditions using the Agilent (B1500A) semiconductor parameter analyzer. The mobility was extracted using the following Supplementary Equation (1)[21]:

$$\mu_{FET} = \frac{dI_{DS}}{dV_G} \cdot \frac{L}{W \cdot C_{ox} \cdot V_{DS}}$$ Supplementary Equation (1)

where L and W are the channel length and width, respectively, C_{ox} is the gate oxide capacitance (11 nF·cm^{-2}), V_{DS} is the source drain voltage, I_{DS} is the source drain current, and V_G is the gate voltage. The linear regime of the transfer characteristics was used to obtain dI_{DS}/dV_G.

Supplementary Figure 18 show electrical properties of the B-doped and N-doped graphene synthesized by B and N ion implantation with fluence of 4×10^{16} atoms/cm^2 respectively. With regard to the B-dope graphene, the sheet resistance values vary from 0.034 to 0.056 kΩ, as shown in Supplementary Figure 18a. Supplementary Figure 18b displays histogram of the field effect mobility distribution for total 30 devices. It is found that the B-doped graphene exhibits good carrier mobilities in the range of 900~1100 cm^{2}V^{-1}s^{-1} for holes and 800~900 cm^{2}V^{-1}s^{-1} for electrons, which are comparable to the carrier mobilities reported for B-doped graphene[22-23]. For the N-doped graphene, the sheet resistance values change from 0.035 to 0.055 kΩ, as shown in Supplementary Figure 18c. Supplementary Figure 18d exhibits histogram of the field effect mobility distribution for total 30 devices. The carrier mobilities (μ_e, μ_h) for the N-doped graphene are estimated as 800~900 cm^2/Vs for electrons and 700~800 cm^2/Vs for holes, respectively, which are comparable to the carrier mobilities reported for N-doped graphene[24-25].

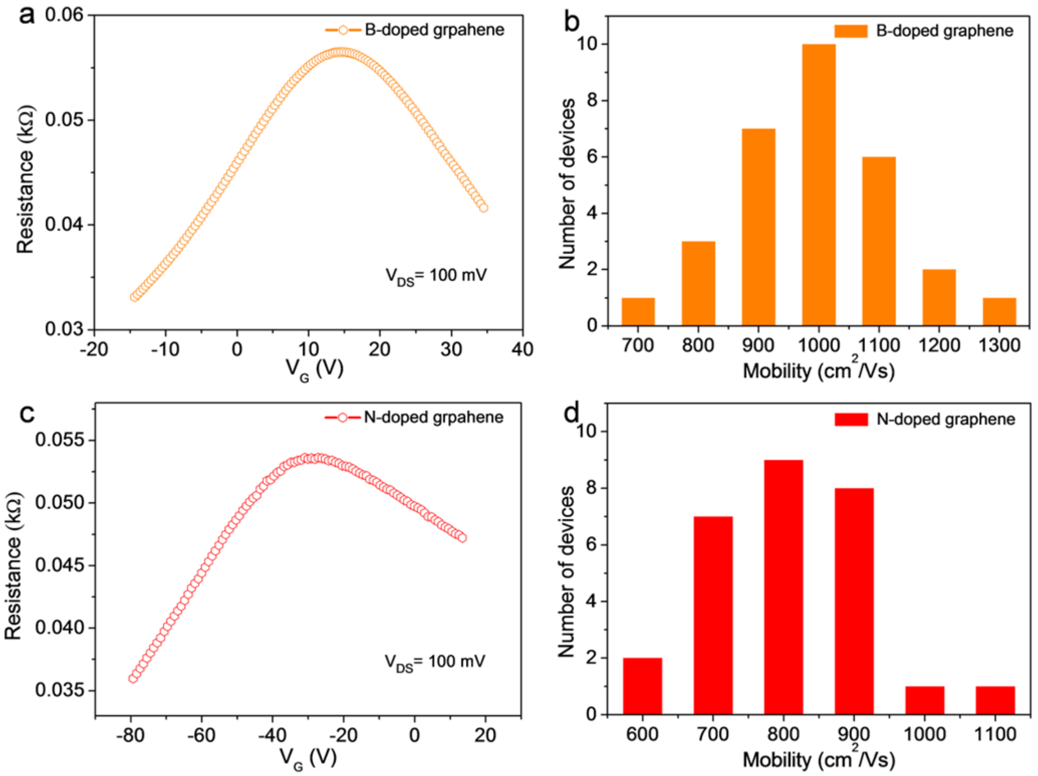

Supplementary Figure 18. Electrical properties of the **a,** B-doped and **c,** N-doped graphene. Histogram of the field effect mobility of **b,** B-doped and **d,** N-doped graphene distribution for total 30 devices. (B-doped and N-doped graphene synthesized by B and N ion implantation with fluence of 4×10^{16} atoms/cm^2)

Supplementary Note X. Responsivity and detectivity of the seamless lateral p-n junctions

The fabricated seamless lateral p-n junctions were transferred onto a highly doped p-type Si substrate coated with 300 nm thick thermal oxide and then Au/Ti (50/10 nm) was deposited by electron beam evaporation to form the source and drain electrodes. Afterwards, another photolithographic step by inductively-coupled plasma (ICP) was used to pattern the p-n junctions to form a photodetector with a channel length of 8

μm and width of 2 μm. The photodetectors were evaluated under ambient conditions using the Agilent (B1500A) semiconductor parameter analyzer and Keithley 4200 semiconductor characterization system. To quantify the performance, two key metrices including the responsivity (R) and detectivity (D^*) reflecting the photodetector sensitivity to incident light were calculated by the following Supplementary Equation (2) & (3)[26-27]:

$$\mathrm{R} = \frac{I_p}{P_{opt}} \quad \text{Supplementary Equation (2)}$$

where I_p, and P_{opt}, are the photocurrent, incident light power, respectively. P_{opt} is the illumination power on the active area (A) of a p-n junction device $P_{opt} = P_0 \times A \times N$. (N represents number of devices)

I_p is photocurrent (4~15.2 nA) as the wavelength is decreased from 1550 nm to 532 nm. The illumination power is 15 mW/cm^2 and the beam spot size is 5 μm.

$$D^* = \frac{R}{\left(2q \cdot I_{dark} / A\right)^{1/2}} \quad \text{Supplementary Equation (3)}$$

where A, q and I_{dark} are the active area, elementary charge and dark current, respectively.

Supplementary Note XI. Photoelectric measurements of the seamless lateral p-n junctions

Supplementary Figure 19 shows the optical micrograph and the SEM image of photodetector constructed on the lateral graphene p-n junction, and the corresponding photocurrent mapping and Raman mapping. In addition to the enhanced photocurrent commonly observed at the interface between electrodes and graphene, the

photocurrent response is also found be localized to the narrow region between the two electrodes, as shown in Supplementary Figures 19a-b. As shown in Supplementary Figure 19b, the photo-responses recorded at point 1 and 4 with opposite polarities is attributed to PV effects at metal-graphene contact, which have been widely reported[28-30]. For the p-n junction region, the polarity of photoelectric outputs at point 2 reversed with that at point 3, which is well consistent with the polarity of the p-n junction. As the position of graphene p-n junction interface indicated by Raman mapping (Supplementary Figure 19d) coincides with the location of photocurrent, it suggests that the generation of photocurrent is due to the formation of p-n junction across the entire B-doped/N-doped area. It should be noted that, if there is no formation of p-n junction, the photoactive areas of the pristine graphene or doped graphene are only confined at the interface of the graphene and metal[28, 31-33], which are quite different from our work.

From the theoretical perspective, there has been no consensus on the physical mechanisms responsible for the light-to-current conversion processes for all graphene p-n junctions so far[28, 33-37]. It is known the two possible photocurrent generation mechanisms, photovoltaic (PV) and photothermoelectric (PTE) effects, are generally believed to contribute to the light-to-current conversion processes in the unbiased graphene p-n junction (The bolometric response of the graphene photodetector can only be observed in biased graphene devices)[38]. Due to the identical photocurrent polarity and comparable photocurrent amplitude induced by PV and PTE effects, it is rather difficult to distinguish which one would be the dominant response mechanism

for photocurrent generated in graphene p-n junction[34-35, 38]. Similar to that of the PV effect, the sign of photothermoelectric (PTE) current in graphene p-n junction is usually unidirectional. However, it should be noted that the sign of photocurrent induced PTE effect can be reversed in the unipolar junction regimes such as pp^+ or nn^-, as described in many literatures[31, 34, 38-41]. The formation of unipolar junction regimes such as pp+ or nn- is very possible if the graphene doping is not strictly uniform (Supplementary Figure 6). In particular, the inhomogeneous doping is easy to occur locally at the interface of p-n junction (Supplementary Figures 19 d-e). The local non-uniform doping is very prone to induce the formation of the unipolar junction regimes such as pp^+ or nn^- near the graphene p-n junction interface. The thermoelectric current can be reversed when the unipolar junction regimes like pp^+ or nn^- are formed, as we observed the highlighted photocurrent with alternating sign in our graphene photodetector built on graphene p-n junction shown in Supplementary Figure 19b.

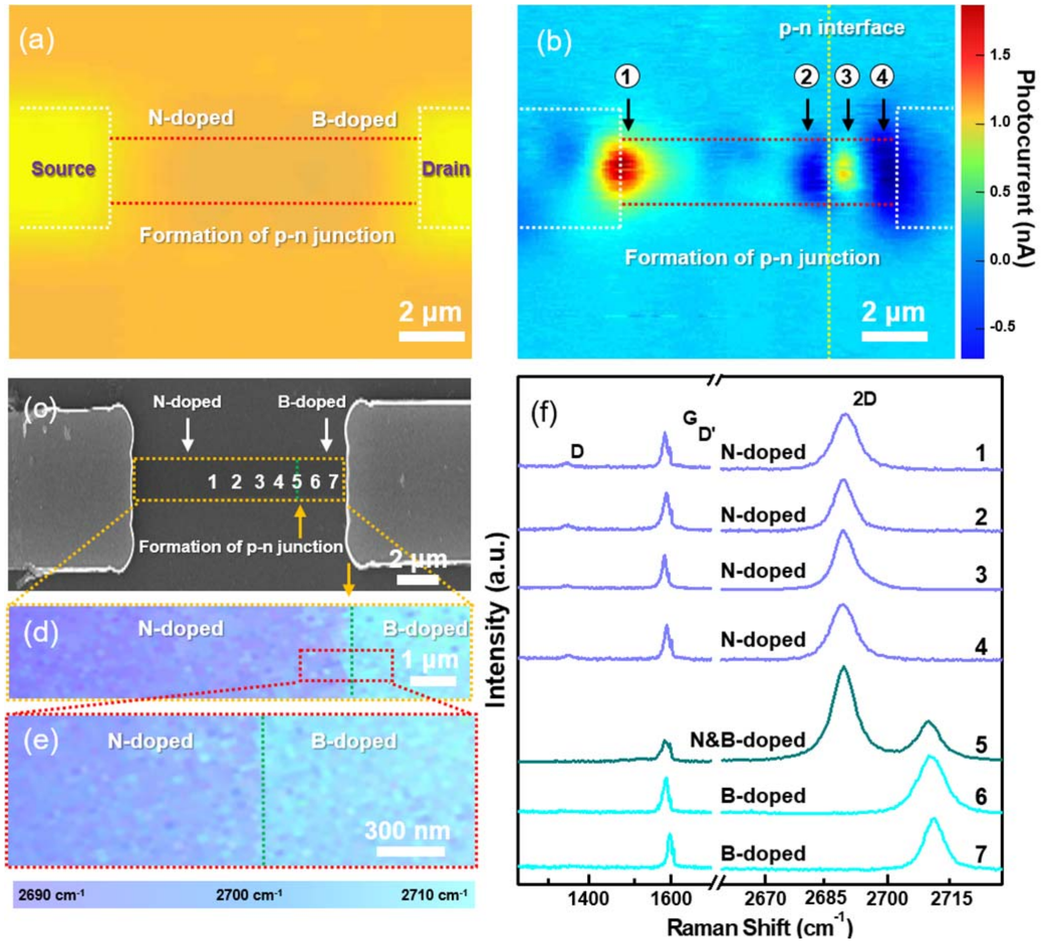

Supplementary Figure 19. a, Optical micrograph of the lateral graphene p-n junction device, and the dotted lines showing the contour of the photodetector. **b,** Photocurrent mapping across the entire lateral graphene p-n junction device. The photoexcitation power is 500 μW and the wavelength is 633 nm. **c,** SEM image of the lateral graphene p-n junction device. **d,** 2D peak mapping of the lateral graphene p-n junction showing the B-doped graphene region (blue) and N-doped graphene region (purple). Mapping is collected from the dashed box in (c). **e,** 2D peak mapping of the junction area of the lateral graphene p-n junction collected from the dashed box in (d). **f,** Raman spectra acquired from seven spots shown in (c), which are distributed across the junction area of the lateral graphene p-n junction device.

When p-n junction is replaced by N-or B- doped graphene, the rectification behavior disappears, and only the linear I_{DS}-V_{DS} behavior is observed, indicating good ohmic contact is formed and no significant Schottky barrier exists at source and drain electrodes, as displayed in Supplementary Figure 20a and Supplementary Figure 20b[42-45].

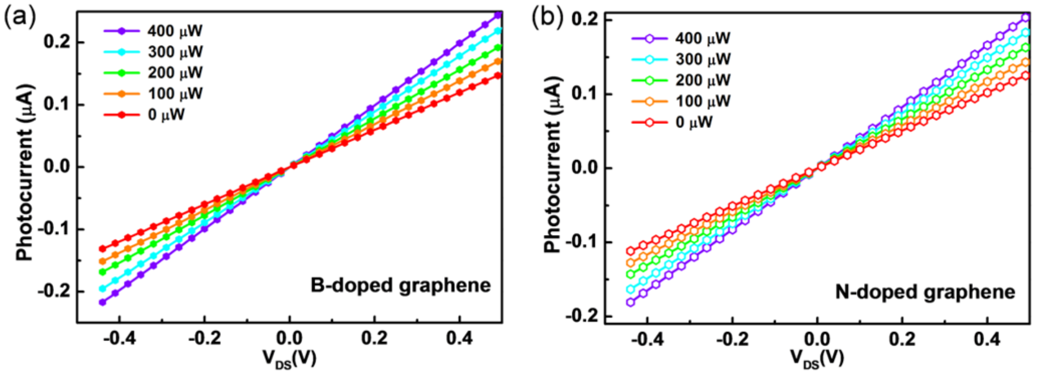

Supplementary Figure 20. Photocurrents I_{DS} at V_G=0 V of the devices of **a,** B-doped graphene synthesized by B ion implantation with a fluence of 4×10^{16} atoms/cm^2 and **b,** N-doped graphene synthesized by N ion implantation with a fluence of 4×10^{16} atoms/cm^2, respectively, as a function of V_{DS} under varying light power. The wavelength is 1550 nm.

The formation of p-n junction is further confirmed by the transfer characteristic curve obtained from FET made of graphene p-n junction, as shown in Supplementary Figure 21. It exhibits two maxima corresponding to the charge neutrality points (Dirac points), separating the whole curve into three regimes labelled p^+-p, p-n and n-n^+, which is the hallmark of a graphene p-n junction[46-48].

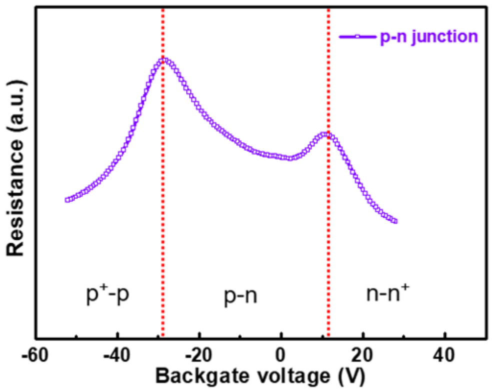

Supplementary Figure 21. Transfer characteristics of graphene p-n junction.

Supplementary Note XII. Performance reproducibility of photodetector device

More than 30 graphene p-n junctions have been tested, as shown in Supplementary Figure 22. From I_{ds}-V_{ds} curves collected from 5 representative devices as displayed in Supplementary Figure 22a, it is observed that the rectification behaviors are obtained in all selected photodetectors constructed on the seamless lateral graphene p-n junction, and the rectification ratios are quite similar. For the photocurrents collected at V_{ds} = 1 V and V_g = 0 V from 30 individual photodetectors, the photocurrent histogram reveals that the majority of photocurrent is in the range from 3.8 to 4.2 nA with an average value of 4 nA, as provided in Supplementary Figure 22b, indicating the photodetector devices constructed on the seamless lateral graphene p-n junction exhibit excellent reproducibility.

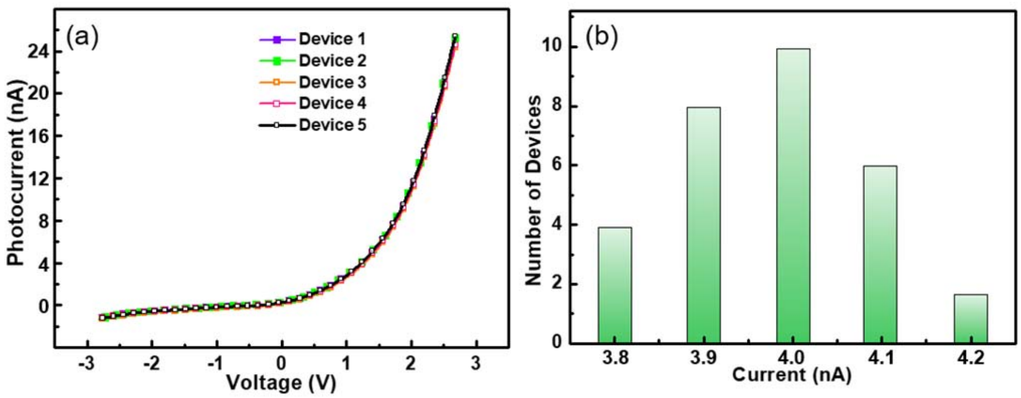

Supplementary Figure 22. **a,** I_{ds}-V_{ds} characteristics of 5 representative photodetectors measured under light illumination with wavelengths of 1550 nm, the light intensity of the light sources is fixed at 15 mW/cm^2. **b,** The distributional histogram of the photocurrent collected at V_{ds} = 1 V and V_g = 0V for 30 devices.

Supplementary Note XIII. Simulation results of lateral and vertical graphene p-n junction diodes

We perform the detailed simulations using Sentaurus TCAD and compare the photo-response behaviors between the lateral graphene p-n junction and the vertical graphene p-n junction. Supplementary Figure 23a and Supplementary Figure 23b show the schematic of the seamless lateral graphene p-n junction and the vertical graphene p-n junction for photodetector application. For the lateral graphene p-n junction, two regions with different doping types are seamlessly merged and the potential barrier at the junction interface of the p-n junction is negligible, as shown in the bottom panel of Supplementary Figure 23a. However, for the vertical graphene p-n junction created by the transfer and stacking of graphene layers with different doping types, the potential barrier exists across the junction interface (the bottom

panel of Supplementary Figure 23b). Using Sentaurus TCAD together with SDE module and SDEVICE module, the electric field distributions located at the junction interfaces in both graphene p-n junctions are obtained, as shown in Supplementary Figure 23c and Supplementary Figure 23d. The maximum electric field distributed in the lateral graphene p-n junction is as high as 9.1×10^3 V/cm, while is much larger than that exists in the vertical graphene p-n junction. Other than the lateral graphene p-n junction, the existence of potential barrier at the junction interface in the vertical graphene p-n junction results in the reduced electric field at the junction interface, which impedes the electron and hole drift, thus resulting in the reduced photoresponse. Using the ray-tracing propagation model in the commercial simulation package named SDEVICE, both dark current and photo current can be obtained on the lateral and vertical graphene p-n junctions, as plotted on the logarithmic scale in Supplementary Figure 23e and Supplementary Figure 23f. It is observed that I-V curves for both graphene p-n junctions show the predominant current-rectifying behavior with a considerable rectification ratio measured either in the dark or under illumination with 637nm laser. However, it is noteworthy that both photo current and dark current of the seamless lateral graphene p-n junction are considerably higher than that collected from the vertical graphene p-n junction, thus leading to the improved photo-response behavior including the responsivity (R) and detectivity (D^*)[26-27].

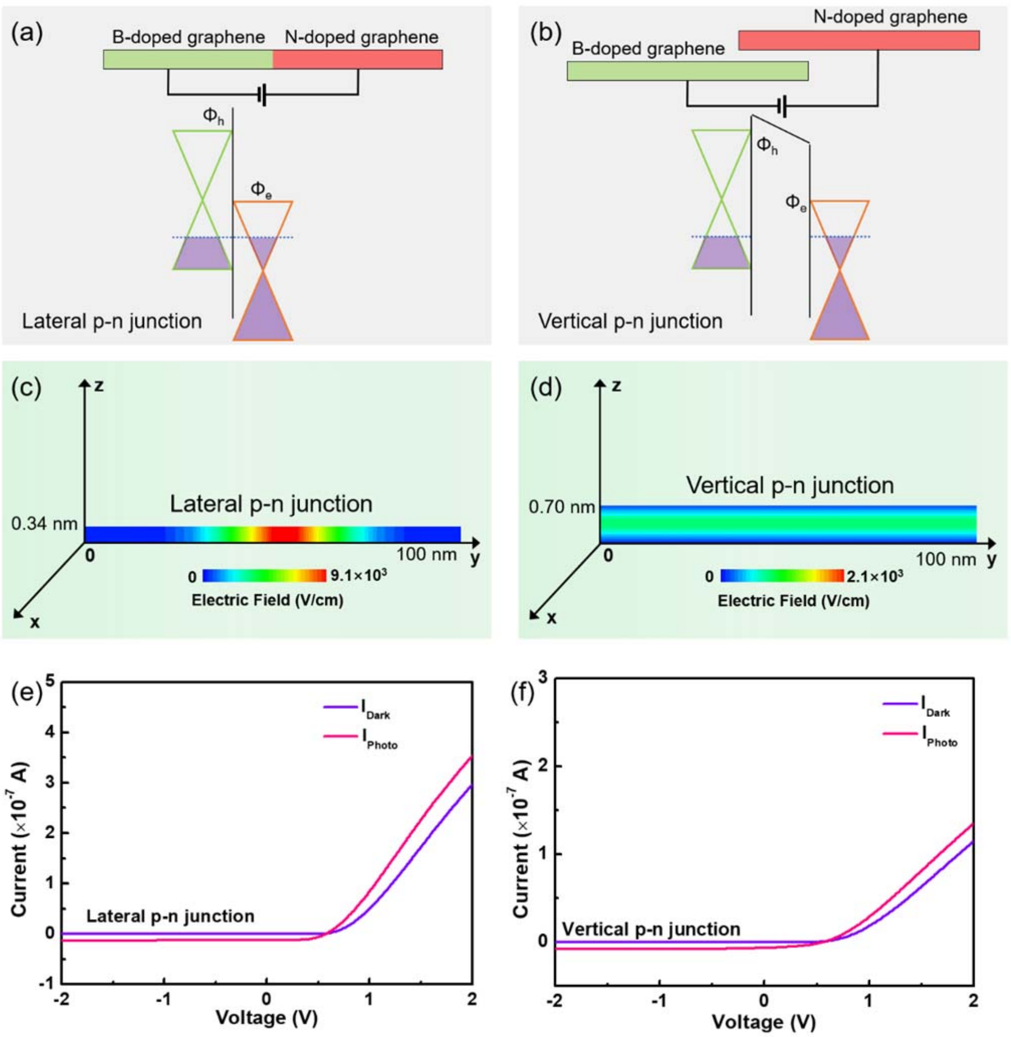

Supplementary Figure 23. a, Schematic structure and energy band diagram of the seamless lateral graphene p-n junction device. **b,** Schematic structure and energy band diagram of the vertical graphene p-n junction device. The simulated electric fields for the **c,** lateral and **d,** vertical graphene p-n junction under 0 V bias. The simulated dark current and photo current for the **e,** lateral and **f,** vertical graphene p-n junction.

Supplementary References

1. Ferrari, A. C. & Basko, D. M. Raman spectroscopy as a versatile tool for studying the properties of graphene. *Nat Nanotechnol.* **8,** 235-246 (2013).

2. Ferrari, A. C. Raman spectroscopy of graphene and graphite: Disorder, electron-phonon coupling, doping and nonadiabatic effects. *Solid State Commun.* **143,** 47-57 (2007).

3. Liu, J. K., Li, Q. Q., Zou, Y., Qian, Q. K., Jin, Y. H., Li, G. H., Jiang, K. L. & Fan, S. S.. The dependence of graphene Raman D-band on carrier density. Nano Lett. **13,** 6170-6175 (2013).

4. Shivaraman, S., Jobst, J., Waldmann, D., Weber, H. B. & Spencer, M. G. Raman spectroscopy and electrical transport studies of free-standing epitaxial graphene: Evidence of an AB-stacked bilayer. *Phys Rev B.* **87,** 195425 (2013).

5. Nair, R. R., Blake1, P., Grigorenko, A. N., Novoselov, K. S., Booth, T. J., Stauber, T., Peres, N. M. R. & Geim, A. K. Fine structure constant defines visual transparency of graphene. *Science* **320,** 1308 (2008).

6. Sun, Z. Z., Raji, A. O., Zhu, Y., Xiang, C. S., Yan, Z., Kittrell, C., Samuel, E. L. G. & Tour, J. M. Large-area bernal-stacked bi-, tri-, and tetralayer Graphene. *ACS Nano.* **6,** 9790-9796 (2012).

7. Han, T. H., Lee, Y. B., Choi, M. R., Woo, S. H., Bae, S. H., Hong, B. H., Ahn, J. H. & Lee, T. W. Extremely efficient flexible organic light-emitting diodes with modified graphene anode. *Nat. Photonics.* **6,** 105-110 (2012).

8. Kim, B. J., Jang, H., Lee, S. K., Hong, B. H., Ahn, J. H. & Cho, J. H. High-performance flexible graphene field effect transistors with ion gel gate dielectrics. *Nano Lett.* **10,** 3464-3466 (2010).

9. Jin, Z., Yao, J., Kittrell, C. & Tour, J. M. Large-scale growth and characterizations

of nitrogen-doped monolayer graphene sheets. *ACS Nano.* **5,** 4112-4117 (2011).

10. Lv, R. *et al*. Ultrasensitive gas detection of large-area boron-doped graphene. *PNAS* **112,** 14527-14532 (2015).
11. Chang, C. K. *et al*. Band gap engineering of chemical vapor deposited graphene by in situ BN doping. *ACS Nano.* **7,** 1333-1341 (2013).
12. Li, X., Wang, H. L., Robinson, J. T., Sanchez, H., Diankov, G. & Dai, H. J. Simultaneous nitrogen doping and reduction of graphene oxide. *J. Am. Chem. Soc.* **36,** 1385-1391 (2012).
13. Wang, G., Zhang, M., Liu, S., Xie, X. M., Ding, G. Q., Wang, Y. Q., Chu, P. K., Gao, H., Ren, W., Yuan, Q. H., Zhang, P. H., Wang, X. & Di. Z. F. Synthesis of layer-tunable graphene: a combined kinetic implantation and thermal ejection approach. *Adv. Funct. Mater.* **25,** 3666-3675 (2015).
14. Li, J., Ji, H. X., Zhang, X., Wang, X. Y., Jin, Z., Wang, D. & Wan, L. J. Controllable atmospheric pressure growth of mono-layer, bi-layer and tri-layer graphene. *Chem. Commun.* **50,** 11012-11015 (2014).
15. Hwang, J., Kim, M., Campbell, D., Alsalman, H. A., Kwak, J. Y., Shivaraman, S., Woll, A. R., Singh, A. K., Hennig, R. G., Gorantla, S., Rummeli, M. H. & Spencer, M. G. van der Waals epitaxial growth of graphene on sapphire by chemical vapor deposition without a metal catalyst. *ACS Nano.* **7,** 385-395 (2013).
16. Feng, X. F., Zhang, L., Ye, Y. F., Han, Y., Xu, Q., Kim, K. J., Ihm, K., Kim, B., Bechtel, H., Martin, M., Guo, J. H. & Zhu, J. F. Engineering the metal-organic

interface by transferring a high-quality single layer graphene on top of organic materials. *Carbon* **87,** 78-86 (2015).

17. Schiros, T. *et al*. Connecting dopant bond type with electronic structure in N-doped graphene. *Nano Lett.* **12,** 4025-4031 (2012).

18. Usachov, D. Y., Fedorov, A. V., Petukhov, A. E., Vilkov, O. Y., Rybkin, A. G., Otrokov, M. M., Arnau, A., Chulkov, E. V., Yashina, L. V., Farjam, M., Adamchuk, V. K., Senkovskiy, B. V., Laubschat, C. & Vyalikh, D. V. Epitaxial B-graphene: large-scale growth and atomic structure. *ACS Nano.* **7,** 7314-7322 (2015).

19. Pop, E., Varshney, V. & Roy, A. K. Thermal properties of graphene: Fundamentals and applications. *MRS Bull.* **37,** 1273-1281 (2012).

20. Wang, G., Zhang, M., Zhu, Y., Ding, G. Q., Jiang, D., Guo, Q. L., Liu, S., Xie, X. M., Chu, P. K., Di, Z. F. & Wang, X. Direct growth of graphene film on germanium substrate. *Sci. Rep.* **3,** 2465 (2013).

21. Liu, G., Stillman, W., Rumyantsev, S., Shao, Q., Shur, M. & Balandin, A. A. Low-frequency electronic noise in the double-gate single-layer graphene transistors. *Appl. Phys. Lett.* **95,** 033103 (2009).

22. Wang, H., Zhou, Y., Wu, D., Liao, L., Zhao, S. L., Peng, H. L. & Liu, Z. F. Synthesis of boron-doped graphene monolayers using the sole solid feedstock by chemical vapor deposition. *Small* **9,** 1316-1320 (2013).

23. Wu, T. R., Shen, H. L., Sun, L., Cheng, B., Liu, B. & Shen, J. C. Nitrogen and boron doped monolayer graphene by chemical vapor deposition using polystyrene, urea and boric acid. *New J. Chem.* **36,** 1385-1391 (2012).

24. Wei, D. C., Liu, Y. Q., Wang, Y., Zhang, H. L., Huang, L. P. & Yu, G. Synthesis of N-doped graphene by chemical vapor deposition and its electrical properties. *Nano Lett*. **9,** 1752-1758 (2009).

25. Jin, Z., Yao, J., Kittrell, C. & Tour, J. M. Large-scale growth and characterizations of nitrogen-doped monolayer graphene sheets. *ACS Nano* **5,** 4112-4117 (2011).

26. Zeng, L. H., Wang, M. Z., Hu, H., Nie, B., Yu, Y. Q., Wu, C, Y., Wang, L., Hu, J. G., Xie, C., Liang, F. X. & Luo, L. B. Monolayer graphene/germanium schottky junction as high-performance self-driven infrared light photodetector. *ACS Appl. Mater. Interfaces*. **5,** 9362-9366 (2013).

27. Liu, X., Gu, L. L., Zhang, Q. P., Wu, J. Y., Long, Y. Z. & Fan, Z. Y. All-printable band-edge modulated ZnO nanowire photodetectors with ultra-high detectivity. *Nat. Commun*. **5,** 4007 (2014).

28. Xia, F. N., Mueller, T., Golizadeh-Mojarad, R., Freitag, M., Lin, Y. M., Tsang, J., Perebeinos, V. & Avouris, P. Photocurrent imaging and efficient photon detection in a graphene transistor. *Nano Lett.* **9,** 1039-1044 (2009).

29. Lee, E. J. H., Balasubramanian, K., Weitz, R. T., Burghard, M. & Kern, K. Contact and edge effects in graphene devices. *Nat. Nanotechnol*. **3,** 486 (2008).

30. Mueller, T., Xia, F. N. & Avouris, P. Graphene photodetectors for high-speed optical communications. *Nat. Photonics* **4**, 297-301 (2010).

31. Park, J. W., Ahn, Y. H. & Ruiz-Vargas, C. Imaging of photocurrent generation and collection in single-layer graphene. *Nano Lett.* **9,** 1742-1746 (2009).

32. Echtermeyer, T. J., Britnell, L., Jasnos, P. K., Lombardo, A., Gorbachev, R.V.,

Grigorenko, A. N., Geim, A. K., Ferrari, A. C. & Novoselov, K. S. Strong plasmonic enhancement of photovoltage in graphene. *Nat. Commun*. **2,** 458 (2011).

33. Freitag, M., Low, T. & Avouris, P. Increased responsivity of suspended graphene photodetectors. *Nano Lett*. **13,** 1644-1648 (2013).

34. Gabor, N. M., Song, J. C. W., Ma, Q., Nair, N. L., Taychatanapat, T., Watanabe, K., Taniguchi, T., Levitov, L. S. & Jarillo-Herrero, P.. Hot carrier-assisted intrinsic photoresponse in graphene. *Science* **334**, 648-652 (2011).

35. Xu, X., Gabor, N. M., Alden, J. S., Zande, A. M. & McEuen, P. L.. Photo-thermoelectric effect at a graphene interface junction. *Nano Lett*. **10**, 562-566 (2010).

36. Sun, D., Aivazian, G., Jones, A. M., Ross, J. S., Yao, W., Cobden, D. & Xu, X. D.. Ultrafast hot-carrier-dominated photocurrent in graphene. *Nat. Nanotechnol.* **7**, 114-118 (2012).

37. Lin, L., Xu, X., Yin, J. B., Sun, J. Y., Tan, Z. J., Koh, A. L., Wang, H., Peng, H. L., Chen, Y. L. & Liu, Z. F. Tuning chemical potential difference across alternately doped graphene p–n junctions for high-efficiency photodetection. *Nano Lett*. **16**, 4094-4101 (2016).

38. Freitag, M., Low, T., & Xia, F. N. Photoconductivity of biased graphene. *Nat. Photonics* **7**, 53-59 (2013).

39. Zhang, Y. W., Zheng, H. M., Wang, Q. Y., Cong, C. X., Hu, L. G., Tian, P. F., Liu, R., Zhang, S. L. & Qiu, Z. J. Competing mechanisms for photocurrent induced at

the monolayer-multilayer graphene junction. *Small* 1800691 (2018).

40. Guo, N., Hu, W. D., Jiang, T., Gong, F., Luo, W. J., Qiu, W. C., Wang, P., Liu, L., Wu, S. W., Liao, L., Chen, X. S. & Lu, W. High-quality infrared imaging with graphene photodetectors at room temperature. *Nanoscale* **8,** 16065-16072 (2016).
41. Jung, M. K., Rickhaus, P., Zihlmann, S., Makk, P. & Schönenberger, C. Microwave photodetection in an ultraclean suspended bilayer graphene p-n junction. *Nano Lett.* **16,** 6988-6993 (2016).
42. Guo, X., Wang, W. H., Nan, H. Y., Yu, Y. F., Jiang, J., Zhao, W. W., Li, J. H., Zafar, Z., Xiang, N., Ni, Z. H., Hu, W. D., You, Y. & Ni, Z. H. High-performance graphene photodetector using interfacial gating. *Optica*. **3,** 1066 (2016).
43. Zhang, C. H., Fu, L., Liu, N., Liu, M. H., Wang, Y. Y. & Liu, Z. F. Synthesis of nitrogen-doped graphene using embedded carbon and nitrogen sources. *Adv. Mater.* **23,** 1020 (2011).
44. Tang, Y. B., Yin, L. C., Yang, Y., Bo, X. H., Cao, Y. L., Wang, H. E., Zhang, W. J., Bello, J., Lee, S. T., Cheng, H. M. & Lee, C. S. Tunable band gaps and p-type transport properties of boron-doped graphenes by controllable ion doping using reactive microwave plasma. *ACS Nano* **6,** 1970 (2012).
45. Sojoudi, H., Baltazar, J., Tolbert, L. M., Henderson, C. L. & Graham, S. Creating graphene p-n junctions using self-assembled monolayers. *ACS Appl. Mater. Interfaces*, **4,** 4781 (2012).
46. Kim, Y. D., Bae, M. H., Seo, J. K., Kim, Y. S., Kim, H., Lee, J. H., Ahn, J. R., Lee, S. W., Chun, S. H. & Park, Y. D. Focused-laser-enabled p-n junctions in graphene

field-effect transistors. *ACS Nano* **7,** 5850-5857 (2013).

47. Wang, S. G., Suzuki, S., Furukawa, K., Orofeo, C. M., Takamura, M. & Hibino, H.. Selective charge doping of chemical vapor deposition-grown graphene by interface modification. *Appl. Phys. Lett.* **103**, 253116 (2013).

48. Cheng, H. C., Shiue, R. J., Tsai, C. C., Wang, W. H. & Chen, Y. T. High-quality graphene p-n junctions via resist-free fabrication and solution-based noncovalent functionalization. *ACS Nano* **5,** 2051-2059 (2011).
